# SK channel upregulation and sex-specific mechanisms drive spinal motoneuron reduced excitability with age

**DOI:** 10.3389/fnagi.2026.1687226

**Published:** 2026-02-04

**Authors:** Ibrahim Abdul Halim, Kalin R. Gerber, Weston B. Gelford, Cierra L. Ellington, Mohamed H. Mousa, Teresa L. Garrett, Sherif M. Elbasiouny

**Affiliations:** 1 Department of Biomedical, Industrial, and Human Factors Engineering, College of Engineering and Computer Science, Wright State University, Dayton, OH, United States; 2 Department of Neuroscience, Cell Biology, and Physiology, Boonshoft School of Medicine, College of Science and Mathematics, Wright State University, Dayton, OH, United States

**Keywords:** aging, motoneuron types, intrinsic excitability, SK channels, spinal motoneurons, weakness

## Abstract

**Introduction:**

Mechanisms underlying age-related weakness are not fully understood, with neuronal mechanisms recently gaining attention. Despite studies on excitatory and inhibitory inputs, conflicting findings on *α*-motoneuron intrinsic excitability and PIC changes highlight a major gap in explaining age-related strength decline.

**Methods:**

Using electrophysiological and immunohistochemical approaches, we present direct assessment of intrinsic excitability, cell size, and ion channel membrane expression in adult spinal *α*-motoneuron types of male and female mice across three age groups: young (3–4 months), middle aged (12–14 months), and old (24–30 months). To account for variability in aging and assess the association with motor function, these physiological and histological parameters were correlated with forelimb and hindlimb grip strength.

**Results:**

Our findings reveal a decline in intrinsic excitability of spinal *α*-motoneurons with aging in both male and female mice, with a more pronounced e!ect in females. Specifically, female motoneurons show increase in rheobase and reduction in firing gain, whereas in males, only firing gain is reduced. Moreover, age-related strength is correlated with *α*- motoneuron excitability; the lower the *α*-motoneuron excitability, the weaker the aged mouse. Notably, fast-type *α*-motoneurons are the most affected by aging-related excitability decline. Further mechanistic analysis indicates sex-specific differences in motoneuron aging: female motoneurons exhibit increased cell capacitance, hyperpolarized resting membrane potential (RMP), and increased expression of SK channels, while male motoneuron show increased expression of SK channels without cell capacitance or RMP alterations. SK increased expression was specific to FF and FI types in male and female mice.

**Discussion:**

These findings reveal sex-specific aging mechanisms in motoneurons, explain women’s higher frailty risk, and identify novel drug targets to counteract age-related muscle weakness and neuromuscular decline in older adults.

## 1 Introduction

Age-related weakness poses a substantial public health concern in the United States, where more than 40% of adults aged 65 and older report difficulty performing daily tasks because of weakness (Seeman et al., 2010). Although this condition is widely acknowledged, its underlying etiology remains poorly understood. Historically, age-related weakness has been attributed primarily to sarcopenia, or the loss of muscle mass. However, multiple studies have shown that muscle mass alone does not directly correlate with strength (Papadakis et al., 1996; Woodhouse et al., 2016; Becker et al., 2015; Delmonico et al., 2009), suggesting that age-related weakness is both multifactorial and more complex than once believed.

Spinal alpha-motoneurons (*α*-MNs), which serve as the final common pathway for motor output, have emerged as a plausible contributor to age-related weakness. Impairments in *α*-MN activation with advancing age may be driven by several mechanisms: (1) reduced excitatory inputs, (2) increased inhibitory inputs, and/or (3) diminished intrinsic motoneuron excitability. Although previous studies have investigated excitatory and inhibitory changes (Castro et al., 2023; Maxwell et al., 2018), age-related alterations in the intrinsic excitability of *α*-MNs remain under studied with a few studies in the literature with limited experimental rigor (Jenkins et al., 2022; Kalmar et al. 2009; Morales et al. 1987). Based on indirect motor unit human recordings, *α*-MN intrinsic excitability is thought to decline with age due to reduced persistent inward currents (PICs) (Wages et al., 2023; Orssatto et al., 2021; Orssatto et al., 2023; Hassan et al., 2021; Guo et al., 2023). However, findings from earlier animal studies have been inconsistent: Kalmar et al. (2009) reported a reduction in FI gain (i.e., decreased MN excitability) with age, whereas Morales et al. (1987) observed a reduction in rheobase (i.e., increased MN excitability) in older animals. These discrepancies have hindered a clear understanding of how aging influences intrinsic MN excitability. Moreover, it remains unknown whether aging affects intrinsic MN excitability similarly in males and females.

To address this gap in knowledge, the present study had three primary objectives: (1) determine whether the intrinsic excitability of spinal *α*-MNs changes with age and sex, and identify specific cell types that may be particularly affected by these changes, (2) Examine whether intrinsic excitability correlates with motor function in aged male and female mice, and (3) elucidate the cellular mechanisms underlying age-related differences in *α*-MN excitability between sexes. We employed intracellular electrophysiological recordings and immunohistochemistry (IHC) to measure intrinsic excitability, cell size, and ion channel membrane expression in adult spinal *α*-MNs from male and female C57BL/6 mice across three age groups: young (3–4 months), middle aged (12–14 months), and old (24–30 months). To account for the variability in aging, these physiological and molecular measurements were correlated with forelimb and hindlimb grip strength.

Our findings reveal that spinal *α*-MNs’ intrinsic excitability declines with aging in both male and female mice, but the effects are more pronounced in females. Specifically, in female MNs, rheobase increased and FI gain decreased with age, whereas in male MNs only FI gain is reduced. Additionally, age-related strength is proportional to *α*-MN excitability, such that the lower α-MN excitability, the weaker the mouse. Notably, fast MNs were the most affected by aging-related changes. Further mechanistic investigation showed that male and female MNs do not age identically. While female MNs exhibit increased cell capacitance, hyperpolarized resting membrane potential (RMP), and increased SK channel expression, male MNs only show increased SK channel expression. By integrating electrophysiological, histological, and functional assessments, this study provides novel insights into the role of *α*-MNs in age-related weakness and identifies novel mechanisms that could serve as effective pharmacological targets to halt the decline in muscle strength with age. Additionally, it advances our understanding of the multifaceted mechanisms underlying weakness in older adults and offers insights into the factors that contribute to older women being more likely to become frail than men (Jenkins et al., 2022).

## 2 Materials and methods

### 2.1 Animals

**Figure 1:**
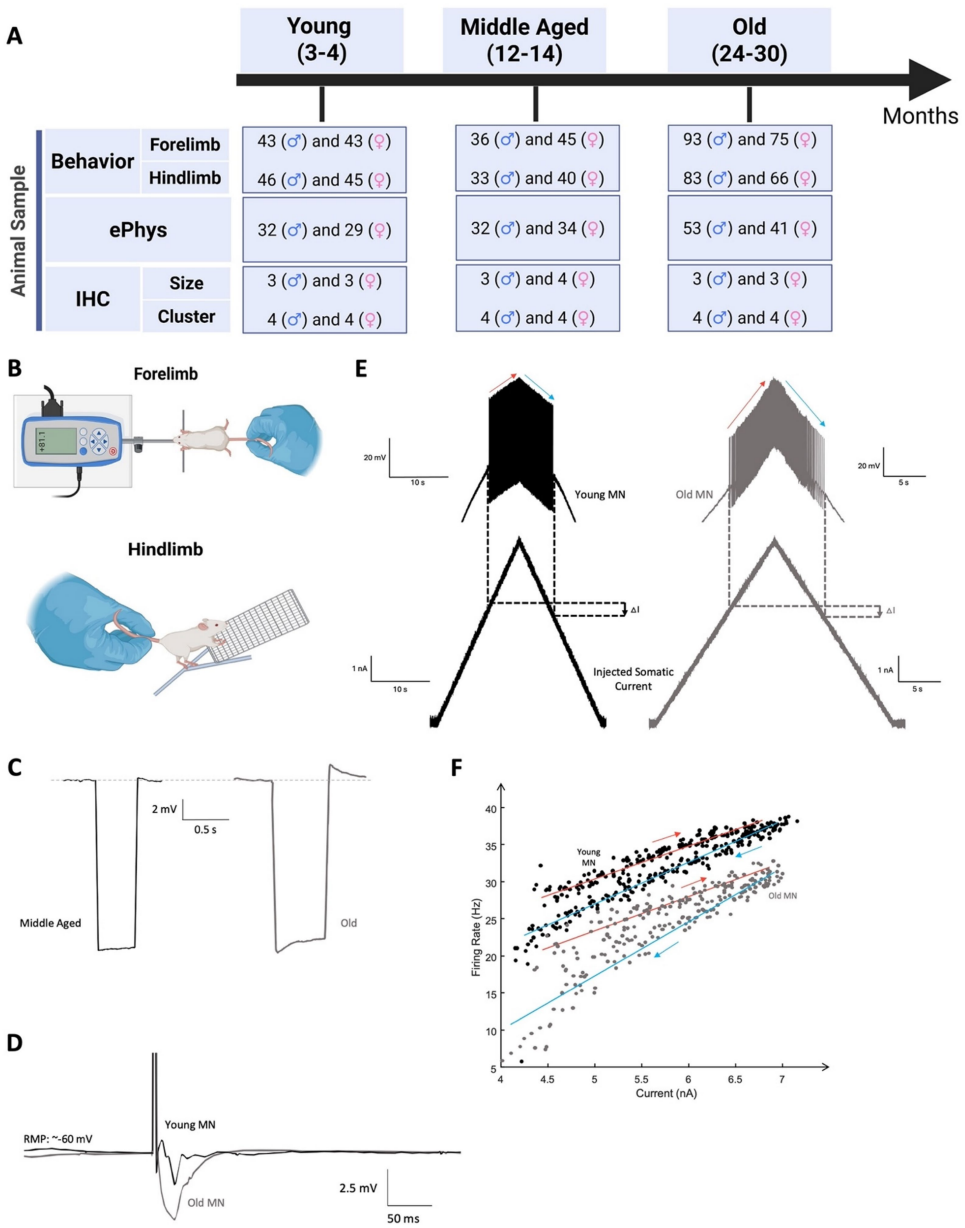
Study experimental design. **(A)** Animal age (in months) and sex distributions for behavioral (forelimb grip strength and hindlimb grip strength), intracellular ePhys, and IHC experiments. **(B)** Demonstration of forelimb and hindlimb grip strength measurements. **(C)** Demonstration of sag ratio traces of middle-aged (black) and old (grey) α-MNs with comparable resting membrane potentials and hyperpolarized to similar potentials. **(D)** Demonstration of AHP traces recorded from young (black) and old (grey) α-MNs with comparable resting membrane potentials. **(E)** Demonstration of repetitive cell firing during triangular current injection recorded from a young (black) and old (gray) α-MN. The red and blue arrows denote the ascending and descending phases, respectively. **(F)** Demonstration of FI relationships of young (black) and old (gray) α-MN. The solid red and blue lines represent the regression lines used to calculate ascending and descending gains for each cell, respectively.

Adult C57BL/6NCrl mice provided by the National Institute on Aging were used in this study. There were three age groups: young (3–4 months), middle aged (12–14 months) and old (24–30 months) (see the experimental design in Figure 1A). For intracellular electrophysiology (ePhys) experiments, a total of 221 adult mice were used with the following sex and age breakdown: 61 young (32 males and 29 females), 66 middle aged (32 males and 34 females) and 94 old (53 males and 41 females). For the cell size IHC experiments, a total of 19 adult mice were used with the following sex and age breakdown: 6 young (3 males and 3 females), 7 middle aged (3 males and 4 females) and 6 old (3 males and 3 females) for determination of cell sizes. For SK cluster analysis by cell type 24 total mice were used: 8 young (4 males and 4 females), 8 middle aged (4 males and 4 females), and 8 old (4 males and 4 females). All surgical and experimental procedures in this study were reviewed and approved by the Institute’s Animal Care and Use Committee (Protocol ID: 2023-0107).

### 2.2 Electrophysiological methodology

#### 2.2.1 *In vitro* tissue preparation

The procedures for the surgical isolation of the sacrocaudal region of the spinal cord followed similar procedures in Mahrous and Elbasiouny (2017). All animals underwent deep anesthesia using Urethane (0.27 g/100 g weight). Supplemental injections were given, if necessary, until the animal was unresponsive to toe/tail pinch and respiration was observably slow and steady. The animal was then securely pinned dorsal side up to a dissecting dish with a steady supply of carbogen (mixture of 95% O2 and 5% CO¬2) via face mask.

Next, the animal’s skin was removed exposing the back muscles. The muscles were cut along both sides of the vertebral column. Starting at the lower thoracic region, a transverse cut was made to expose the spinal cord. Once exposed, modified artificial cerebrospinal fluid (mACSF) aerated with carbogen was steadily dripped on the surgical site at a rate of 4–5 mL/min. The composition of mACSF followed (Mahrous and Elbasiouny, 2017) and contained the following (in millimolar): 118 NaCl, 3 KCl, 1.3 MgSO4, 5 MgCl2, 1.4 NaH2PO4, 1.5 CaCl2, 24 NaHCO3, and 25 glucose. The vertebral column was then carefully cut down the spinal cord as far as possible to expose the sacrocaudal roots. At around L3, the spinal cord was transected, and the dura mater was longitudinally cut. The cord was gently lifted to cut the spinal root ends and freeing the spinal cord from the vertebral column. The spinal cord was then carefully transported to a carbogen aerated mACSF filled petri dish and the animal was decapitated (an AMVA-approved method of euthanasia). In the petri dish, both the dorsal and ventral spinal roots in the S1-C02 segments were separated and freed from the spinal cord and neighboring roots. The cord was then transected at around L6, and the S1-C02 segments were transferred to a recording chamber. The recording chamber was continuously perfused with carbogen aerated normal artificial cerebrospinal fluid (nACSF) at a rate of 2.5–3 mL/min. The nACSF solution was composed of (in millimolar): 128 NaCl, 3 KCl, 1.5 MgSO4, 1 NaH2PO4, 2.5 CaCl2, 22 NaHCO3, and 12 glucose, adopted from Mahrous and Elbasiouny (2017). In the recording chamber, the spinal cord was pinned ventral side up and the spinal roots were placed on bipolar electrodes. The roots were covered with petroleum jelly to aid in cord stability and root moisture. Upon the completion of this process, the spinal cord was incubated for approximately 1 h recovery period before the start of intracellular MN recordings.

### 2.3 Intracellular recordings

#### 2.3.1 Motoneuron identification

Current clamp MN recordings were made with sharp glass micropipettes. Sharp glass micropipettes were pulled from 2 mm outer-diameter thick-walled borosilicate capillary glass (A-M Systems, Sequim, WA, USA, 604000) using a P97 micropipette puller (Sutter Instrument, Novato, CA). Micropipettes were then filled with 3 M KC2H3O2 and 100 mM KCl. Next, a silver chloride electrode was placed inside the micropipette for intracellular MN recording. Micropipettes typically had a measured resistance range of 36–43 MΩ. A 2660 model micro positioner (2,660; David Kopf Instruments, Tujunga, CA) was used to vertically advance the micropipette into the ventral horn of the spinal cord until a MN was identified. All MNs were verified by an antidromic AP through the stimulation of the mounted ventral roots. Once MNs were stabilized, they were verified by a ≤ −55 mV resting membrane potential and a ≥ 50 mV antidromic AP height. Following our earlier work (Deutsch and Elbasiouny, 2024), All intracellular recordings were performed using the Axoclamp-2A amplifier (Molecular Devices, CA) running in discontinuous current clamp (DCC) mode (6–8 kHz) with consideration of the results and guidance from Manuel (2021). All collected intracellular recordings were digitized using the Power 1,401-3A CED board (Cambridge Electronic Design, United Kingdom). The Spike2 software (version 8.23, Cambridge Electronic Design) was utilized for the acquired recordings and stored for offline data analysis.

#### 2.3.2 Motoneuron excitability measurement

Repetitive firing in MNs was evoked by injecting a triangular ramp of current with a steady slope of 0.5 nA/s. Following that, MN firing frequency was calculated from the evoked repetitive firing throughout the entire triangular ramp. The firing frequency was plotted versus injected current to graph the frequency-current (FI) relationship of the cell and calculate the ascending and descending FI gains from the slopes of the FI ascending and descending segments (see Figures 1E,F for firing and FI data of a young and old cell). Another excitability measurement collected was rheobase, which was measured by injecting the MN with 0.1 nA increments of 50 ms depolarizing current pulses until a single AP was elicited. The sag ratio was quantified according to the method described by Delestrée et al. (2014) and Manuel et al. (2005). Sag potentials arise from activation and deactivation of the hyperpolarization-activated cation (HCN) channels mediating I_h_, the principal current activated by hyperpolarization and deactivated by depolarization in spinal MNs (Manuel et al., 2005; Kiehn et al., 2000; Takahashi, 1990), which has been shown to play a role in regulating the excitability of brainstem and spinal neurons (Reedich et al., 2023; Sharples and Miles, 2021; Bayliss et al., 1994). Sag ratios were compared among cells that were hyperpolarized to similar membrane potentials and were quantified using a series of incremental hyperpolarizing current pulses that lowered the membrane potential up to ~10 mV below rest (i.e., within the same voltage range in which AHP activation occurs, Figure 1C).

#### 2.3.3 Measurement of action potential properties

Impaled MNs were injected with 5 consecutive pulses of depolarizing current with 1.1 s intervals. The evoked APs were averaged and analyzed for the following properties: max AP rising slope (V/s), max AP falling slope (V/s), AP height (mV), AP width (ms), AHP amplitude (mV), AHP duration (ms), and relative SK conductance (rGSK). AP and AHP properties were calculated and shown in Table 1. The max AP rising and falling slopes were measured as the maximum slope values during the AP depolarization and repolarization phases, respectively. AP height was measured as the voltage value from the resting membrane potential to the highest voltage point reached by the AP. AP width was measured as the time between the AP rising and falling phases. AHP amplitude was measured as the voltage difference between the deepest point of the AHP and the resting membrane potential. AHP duration was measured as the time between the start of the AHP to the voltage point at which the AHP returned to resting membrane potential. GSK was measured by injecting the MN with the 5 consecutive pulses at 3 different MN membrane potentials following the methodology of Li and Bennett (2007). Finally, rGSK was measured by dividing the cell GSK by the cell input conductance. This property normalizes GSK to the cell size.

**Table 1:** Electrophysiological properties of α-MNs.

ePhys parameter	Mean ± SD	One-way ANOVA	Pearson correlation		Regression slope	# of MNs	# of animals	Figures
RMP (mV)	YNG: −67.99 ± 7.34 MA: −66.97 ± 7.59 OLD: −69.63 ± 7.30	YNG-MA: *p* = 0.593 MA-OLD: *p* = 0.012 YNG-OLD: *p* = 0.206 *t*-statistic: 4.280	All: *p* = 0.024 ♀: *p* = 0.07 ♂: *p* = 0.165	All: *r* = −0.120 ♀: *r* = −0.133 ♂: *r* = −0.107	All: *m* = −0.091 ♀: *m* = −0.102 ♂: *m* = −0.080	YNG: 95 (♀: 46, ♂: 49) MA: 108 (♀: 55, ♂: 53) OLD: 153 (♀: 86, ♂: 67)	YNG: 60 (♀: 29, ♂: 31) MA: 61 (♀: 33, ♂: 28) OLD: 82 (♀: 46, ♂: 36)	–
Max rising slope (V/s)	YNG: 297.80 ± 112.23 MA: 289.67 ± 149.77 OLD: 307.70 ± 114.89	YNG-MA: *p* = 0.890 MA-OLD: *p* = 0.489 YNG-OLD: *p* = 0.819 *t*-statistic: 0.665	All: *p* = 0.647 ♀: *p* = 0.462 ♂: *p* = 0.126	All: *r* = 0.024 ♀: *r* = −0.054 ♂: *r* = 0.118	All: *m* = 0.311 ♀: *m* = −0.750 ♂: *m* = 1.357	YNG: 95 (♀: 46, ♂: 49) MA: 109 (♀: 56, ♂: 53) OLD: 153 (♀: 86, ♂: 67)	YNG: 60 (♀: 29, ♂: 31) MA: 61 (♀: 33, ♂: 28) OLD: 82 (♀: 46, ♂: 36)	–
Max falling slope (V/s)	YNG: −276.93 ± 110.76 MA: −273.73 ± 193.08 OLD: −281.08 ± 111.54	YNG-MA: *p* = 0.986 MA-OLD: *p* = 0.910 YNG-OLD: *p* = 0.973 *t*-statistic: 0.088	All: *p* = 0.966 ♀: *p* = 0.316 ♂: *p* = 0.207	All: *r* = 0.002 ♀: *r* = 0.074 ♂: *r* = −0.097	All: *m* = 0.032 ♀: *m* = 1.212 ♂: *m* = −1.134	YNG: 95 (♀: 46, ♂: 49) MA: 109 (♀: 56, ♂: 53) OLD: 153 (♀: 86, ♂: 67)	YNG: 60 (♀: 29, ♂: 31) MA: 61 (♀: 33, ♂: 28) OLD: 82 (♀: 46, ♂: 36)	–
Spike height (mV)	YNG: 59.32 ± 15.33 MA: 59.38 ± 10.32 OLD: 62.94 ± 10.70	YNG-MA: *p* = 0. 999 MA-OLD: *p* = 0.052 YNG-OLD: *p* = 0.061 *t*-statistic: 3.888	All: *p* = 0.000 ♀: *p* = 0.000 ♂: *p* = 0.071	All: *r* = 0.214 ♀: *r* = 0.311 ♂: *r* = 0.138	All: *m* = 0.212 ♀: *m* = 0.343 ♂: *m* = 0.125	YNG: 86 (♀: 42, ♂: 44) MA: 98 (♀: 53, ♂: 46) OLD: 160 (♀: 70, ♂: 90)	YNG: 51 (♀: 25, ♂: 26) MA: 52 (♀: 27, ♂: 25) OLD: 80 (♀: 36, ♂: 44)	–
Spike width (ms)	YNG: 3.78 ± 1.65 MA: 4.27 ± 2.54 OLD: 3.81 ± 1.86	YNG-MA: *p* = 0 0.212 MA-OLD: *p* = 0.172 YNG-OLD: *p* = 0.996 *t*-statistic: 2.000	All: *p* = 0.737 ♀: *p* = 0.970 ♂: *p* = 0.615	All: *r* = −0.018 ♀: *r* = −0.003 ♂: *r* = −0.039	All: *m* = −0.004 ♀: *m* = −0.001 ♂: *m* = −0.007	YNG: 95 (♀: 46, ♂: 49) MA: 109 (♀: 56, ♂: 53) OLD: 153 (♀: 86, ♂: 67)	YNG: 60 (♀: 29, ♂: 31) MA: 61 (♀: 33, ♂: 28) OLD: 82 (♀: 46, ♂: 36)	–
ΔI (nA)	YNG: 0.18 ± 0.52 MA: 0.51 ± 0.81 OLD: 0.44 ± 0.63	YNG-MA: *p* = 0.099 MA-OLD: *p* = 0.897 YNG-OLD: *p* = 0.099 *t*-statistic: 2.920	All: *p* = 0.075 ♀: *p* = 0.535 ♂: *p* = 0.114	All: *r* = 0.154 ♀: *r* = 0.079 ♂: *r* = 0.189	All: *m* = 0.009 ♀: *m* = 0.004 ♂: *m* = 0.013	YNG: 44 (♀: 25, ♂: 19) MA: 27 (♀: 13, ♂: 14) OLD: 64 (♀: 26, ♂: 38)	YNG: 28 (♀: 15, ♂: 13) MA: 21 (♀: 10, ♂: 11) OLD: 46 (♀: 20, ♂: 26)	–
Ascending gain (Hz/nA)	YNG: 9.65 ± 5.43 MA: 6.37 ± 3.06 OLD: 6.34 ± 3.48	YNG-MA: *p* = 0.004 MA-OLD: *p* = 1.000 YNG-OLD: *p* = 0.000 *t*-statistic: 9.722	All: *p* = 0.000 ♀: *p* = 0.005 ♂: *p* = 0.018	All: *r* = −0.308 ♀: *r* = −0.343 ♂: *r* = −0.279	All: *m* = −0.127 ♀: *m* = −0.115 ♂: *m* = −0.133	YNG: 46 (♀: 26, ♂: 20) MA: 28 (♀: 14, ♂: 14) OLD: 65 (♀: 27, ♂: 38)	YNG: 30 (♀: 16, ♂: 14) MA: 22 (♀: 11, ♂: 11) OLD: 47 (♀: 21, ♂: 26)	Figures 4A,B
Descending gain (Hz/nA)	YNG: 11.78 ± 6.82 MA: 7.71 ± 4.08 OLD: 7.01 ± 3.65	YNG-MA: *p* = 0.003 MA-OLD: *p* = 0.814 YNG-OLD: *p* = 0.000 *t*-statistic: 12.612	All: *p* = 0.000 ♀: *p* = 0.007 ♂: *p* = 0.001	All: *r* = −0.369 ♀: *r* = −0.334 ♂: *r* = −0.398	All: *m* = −0.186 ♀: *m* = −0.144 ♂: *m* = −0.227	YNG: 44 (♀: 25, ♂: 19) MA: 28 (♀: 13, ♂: 14) OLD: 64 (♀: 26, ♂: 38)	YNG: 28 (♀: 15, ♂: 13) MA: 21 (♀: 10, ♂: 11) OLD: 46 (♀: 20, ♂: 26)	Figures 4C,D
Rheobase (nA)	YNG: 1.80 ± 1.29 MA: 1.49 ± 1.30 OLD: 2.09 ± 1.44	YNG-MA: *p* = 0.391 MA-OLD: *p* = 0.014 YNG-OLD: *p* = 0.362 *t*-statistic: 4.037	All: *p* = 0.057 ♀: *p* = 0.039 ♂: *p* = 0.459	All: *r* = 0.122 ♀: *r* = 0.190 ♂: *r* = 0.067	All: *m* = 0.017 ♀: *m* = 0.022 ♂: *m* = 0.010	YNG: 67 (♀: 34, ♂: 33) Outliers: 1 ♀ cell MA: 69 (♀: 37, ♂: 32) OLD: 106 (♀: 47, ♂: 59)	YNG: 43 (♀: 22, ♂: 21) MA: 42 (♀: 21, ♂: 21) OLD: 59 (♀: 28, ♂: 31)	Figures 4E,F
Gin (μS)	YNG: 0.0379 ± 0.012 MA: 0.0377 ± 0.010 OLD: 0.0377 ± 0.011	YNG-MA: *p* = 0.974 MA-OLD: *p* = 0.997 YNG-OLD: *p* = 0.985 *t*-statistic: 0.025	All: *p* = 0.721 ♀: *p* = 0.943 ♂: *p* = 0.846	All: *r* = 0.017 ♀: *r* = 0.005 ♂: *r* = 0.013	All: *m* = 0.000 ♀: *m* = 0.000 ♂: *m* = 0.000	YNG: 110 (♀: 55, ♂: 55) Outliers: 1 ♂ cell MA: 144 (♀: 67, ♂: 77) OLD: 201 (♀: 90, ♂: 111) Outliers: 2 ♀, 2 ♂	YNG: 60 (♀: 28, ♂: 32) MA: 66 (♀: 34, ♂: 32) OLD: 91 (♀: 40, ♂: 51)	Figures 7A,B
Cell capacitance (nF)	YNG: 0.0418 ± 0.030 MA: 0.0299 ± 0.050 OLD: 0.0492 ± 0.073	YNG-MA: *p* = 0.252 MA-OLD: *p* = 0.009 YNG-OLD: *p* = 0.539 *t*-statistic: 4.422	All: *p* = 0.058 ♀: *p* = 0.039 ♂: *p* = 0.519	All: *r* = 0.091 ♀: *r* = 0.147 ♂: *r* = 0.042	All: *m* = 0.001 ♀: *m* = 0.001 ♂: *m* = 0.000	YNG: 106 (♀: 51, ♂: 55) Outliers: 1 ♀ cell MA: 134 (♀: 61, ♂: 73) OLD: 192 (♀: 86, ♂: 106)	YNG: 59 (♀: 27, ♂: 32) MA: 66 (♀: 34, ♂: 32) OLD: 91 (♀: 40, ♂: 51)	Figures 7C,D
AHP amplitude (mV)	YNG: 2.26 ± 1.47 MA: 1.89 ± 1.34 OLD: 2.38 ± 1.35	YNG-MA: *p* = 0.136 MA-OLD: *p* = 0.015 YNG-OLD: *p* = 0.814 *t*-statistic: 4.060	All: *p* = 0.17 ♀: *p* = 0.909 ♂: *p* = 0.06	All: *r* = 0.073 ♀: *r* = 0.009 ♂: *r* = 0.141	All: *m* = 0.010 ♀: *m* = 0.001 ♂: *m* = 0.020	YNG: 92 (♀: 46, ♂: 46) MA: 109 (♀: 55, ♂: 54) OLD: 150 (♀: 72, ♂: 78)	YNG: 57 (♀: 29, ♂: 28) MA: 61 (♀: 33, ♂: 28) OLD: 79 (♀: 37, ♂: 42)	Figures 9A,B
AHP duration (ms)	YNG: 54.62 ± 34.63 MA: 70.78 ± 53.94 OLD: 72.69 ± 66.86	YNG-MA: *p* = 0.104 MA-OLD: *p* = 0.960 YNG-OLD: *p* = 0.040 *t*-statistic: 3.268	All: *p* = 0.02 ♀: *p* = 0.045 ♂: *p* = 0.161	All: *r* = 0.124 ♀: *r* = 0.153 ♂: *r* = 0.105	All: *m* = 0.712 ♀: *m* = 0.804 ♂: *m* = 0.648	YNG: 92 (♀: 46, ♂: 46) MA: 109 (♀: 55, ♂: 54) OLD: 150 (♀: 72, ♂: 78)	YNG: 57 (♀: 29, ♂: 28) MA: 61 (♀: 33, ♂: 28) OLD: 79 (♀: 37, ♂: 42)	Figures 9C,D
rGSK	YNG: 0.0959 ± 0.0816 MA: 0.0905 ± 0.107 OLD: 0.1316 ± 0.165	YNG-MA: *p* = 0.958 MA-OLD: *p* = 0.042 YNG-OLD: *p* = 0.129 *t*-statistic: 3.545	All: *p* = 0.013 ♀: *p* = 0.361 ♂: *p* = 0.011	All: *r* = 0.137 ♀: *r* = 0.072 ♂: *r* = 0.072	All: *m* = 0.002 ♀: *m* = 0.001 ♂: *m* = 0.003	YNG: 79 (♀: 38, ♂: 41) Outliers: 1 ♀ cell MA: 102 (♀: 52, ♂: 50) OLD: 147 (♀: 72, ♂: 75)	YNG: 52 (♀: 24, ♂: 28) MA: 60 (♀: 33, ♂: 27) OLD: 80 (♀: 39, ♂: 41)	Figures 9E,F
Sag ratio	YNG: 1.0424 ± 0.033 MA: 1.0381 ± 0.036 OLD: 1.0498 ± 0.039	YNG-MA: *p* = 0.677 MA-OLD: *p* = 0.021 YNG-OLD: *p* = 0.250 *t*-statistic: 3.778	All: *p* = 0.026 ♀: *p* = 0.214 ♂: *p* = 0.057	All: *r* = 0.112 ♀: *r* = 0.093 ♂: *r* = 0.131	All: *m* = 0.000 ♀: *m* = 0.000 ♂: *m* = 0.000	YNG: 96 (♀: 47, ♂: 49) MA: 117 (♀: 57, ♂: 60) OLD: 178 (♀: 76, ♂: 104)	YNG: 56 (♀: 26, ♂: 30) MA: 63 (♀: 33, ♂: 30) OLD: 90 (♀: 39, ♂: 51)	Figures 9G,H

### 2.4 Immunohistochemistry methodology

#### 2.4.1 Tissue preparation

All animals were anesthetized with Euthasol (pentobarbital sodium and phenytoin sodium) solution. Anesthetization was confirmed with non-response to a bilateral lower extremity pain reflex test. Animals were transcardially perfused with a vascular rinse solution (0.01 M phosphate buffer with 0.8% NaCl, 0.025% KCl, and 0.05% NaHCO3, pH 7–8) followed by 4% paraformaldehyde in 0.1 M phosphate buffer, pH 7–8. Total dorsal laminectomy was performed immediately following fixation with complete extraction of the spinal cord. Cords were post-fixed in a 4% paraformaldehyde solution for 2 h and then stored in 15% sucrose at 4 °C. Lumbar sections L3-L6 were sectioned transversely at 45 μm and stored in a cryoprotective solution (0.1 M phosphate buffer, 30% sucrose, 1% polyvinylpyrrolidone, 30% ethylene glycol, pH 7–8).

Two separate sets of animals were assessed with IHC using the following protocols. Sections were rinsed with PBS-T (0.01 M PBS containing 0.1% Triton-X, pH 7.3) placed in citrate buffer and heated to 95 °C for 20 min, then blocked with normal horse serum (10% in PBS-T). One set of animals was labeled with the antibodies for calcium-activated potassium channel subfamily N member 2 (SK2; 1:1000 dilution; goat, Abcam, AB99457), calcium-activated potassium channel subfamily N member 3 (SK3; 1:1000 dilution; rabbit, Millipore, AB5350), Choline acetyltransferase enzyme (ChAT, 1:100 dilution, mouse, Novus, NBP1-30052) and neuronal nuclei (NeuN, 1:300 dilution, guinea-pig, Millipore, ABN90). *α*-MNs with SK2 labeling represent fast cells, while SK3 + α-MNs with SK3 labeling represent slow cells (Deardorff et al., 2013). These animals were used to gather LCA data for fast and slow cells (See section 3.5). The second set of animals was used to type α-MNs using previous methodology (Garrett et al., 2025). Sections were incubated with SK2, SK3 and Osteopontin (OPN, 1:1000 dilution R&D systems, AF808). All antibodies were diluted with PBS-T. Sections floating in primary antibody solutions were incubated at 4 °C overnight. Fab Fragment donkey anti-goat (Jackson ImmunoResearch, 705-007-003) was used as an additional blocking step between SK2 and OPN incubation. Immunofluorescence was achieved through labeling with rabbit, goat, guinea-pig, and mouse-specific secondary antibodies conjugated to Cy3 and Alexa Fluors 488, 405, and 647 (Jackson ImmunoResearch; catalog 711-165-152, 715-545-150, 715-605-150, 706-475-148, 705-545-147, 705-605-147, 7015-475-147). Secondary antibodies were diluted 1:100 in PBS-T, and the free-floating sections were incubated for approximately 3 h. Sections were washed in a cupric sulfate buffer for 1 h then mounted onto glass slides and cover-slipped with Vectashield mounting medium. Slides were stored at 4 °C until imaging.

**Figure 2:**
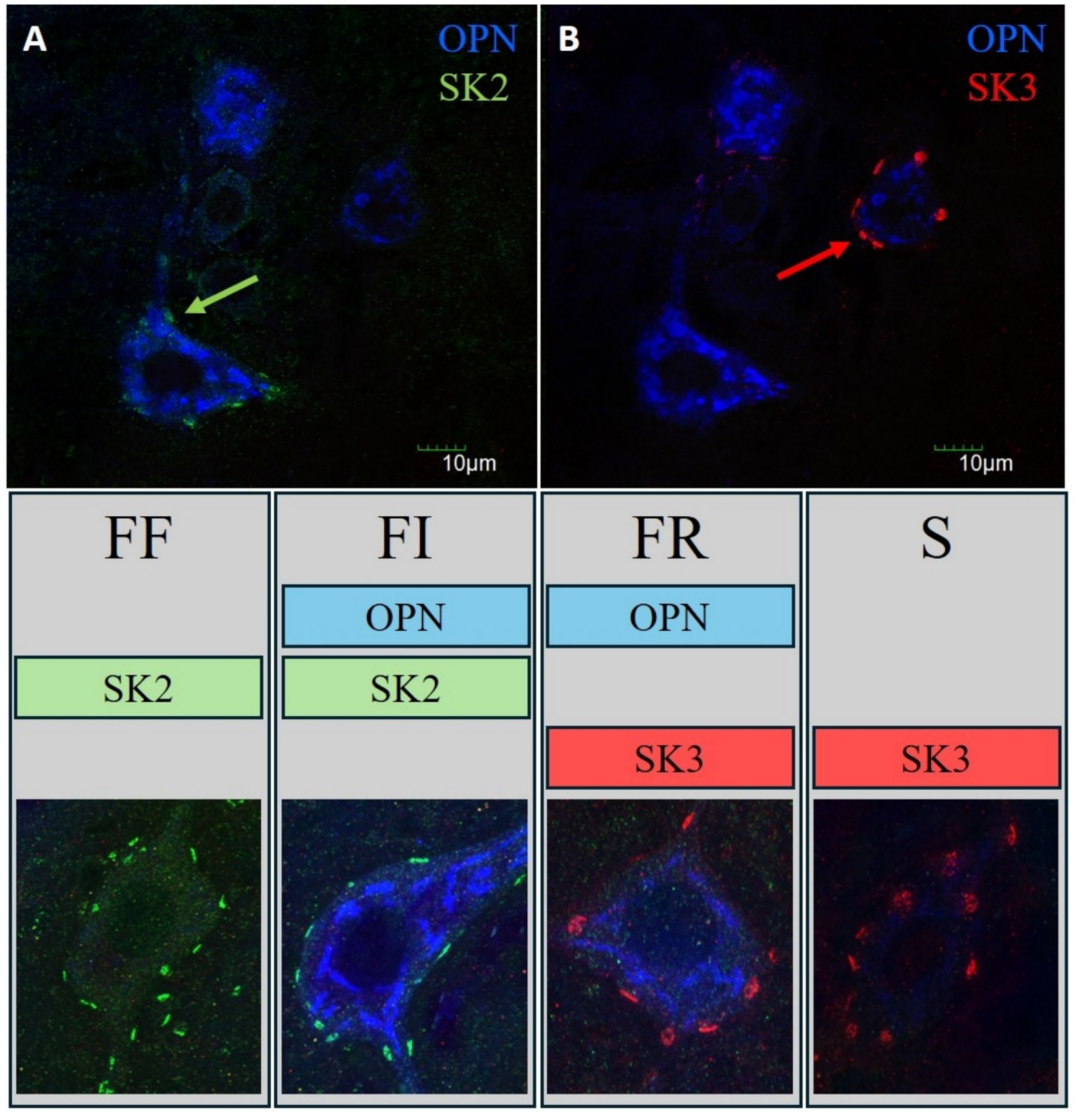
MN subtypes identified by OPN immunolabeling and SK channel expression **(A,B)** Representative images of α-MNs; OPN (blue) co-labeled for SK2 [green, **(A)**] or SK3 [red, **(B)**]. Arrows indicate distinct SK2 (green arrow) and SK3 (red arrow) clustering on the soma membrane. Lower panels: MN subtypes were identified based on OPN immunoreactivity and SK channel expression patterns. FF and FI MNs are SK2-positive, while FF MNs are OPN-negative, and FI MNs are OPN-positive. FR and S MNs are SK3-positive, while FR MNs are OPN-positive, and S MNs are OPN-negative. Each cell type displays distinct SK channel clustering consistent with its classification. Scale bars: 10 μm.

Per the methodology of Garrett et al. (2025), *α*-MNs were classified into S, FR, FI, and FF types using SK2, SK3, and OPN co-labeling. SK2 allows us to determine fast cells and SK3 determines slow cells while OPN further parses apart the different cell types. Figure 2 describes how each α-MN type is determined. Following prior literature (Highlander et al., 2025), the MN typing algorithm was calibrated via empirical comparison with manually classified cells. Decision cut-offs for assigning positive or negative labels were iteratively adjusted to achieve concordance with manual classifications for cells exhibiting unambiguous morphological characteristics. Thresholds were subsequently fine-tuned to optimize classification of cells with ambiguous features, such that the resulting cell size distributions demonstrated the expected progressive increase from S to FI to FR to FF types. In addition, sensitivity analysis was conducted on the SK cluster analysis ensuring precise measurements. Precisely, SK cluster number and volume changes are preserved with age (S1).

**Figure 3:**
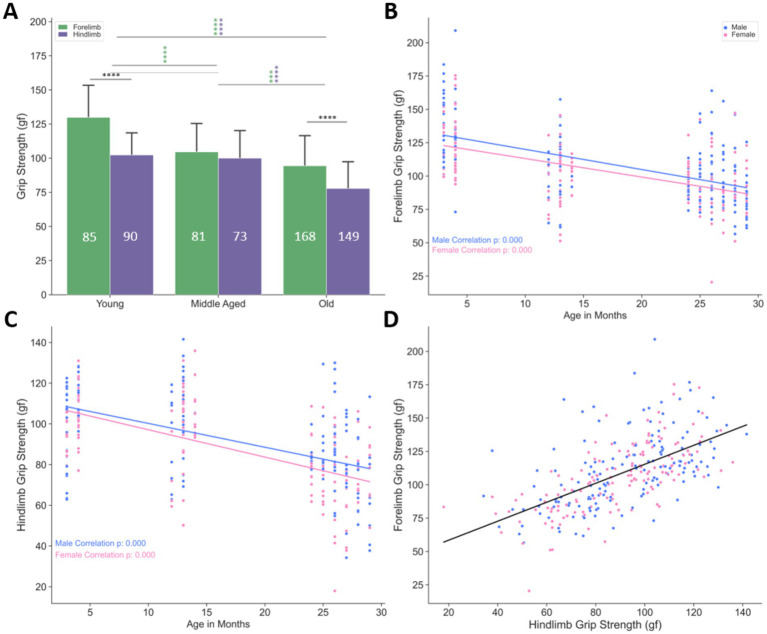
Grip strength decreases with age in C57BL/6 mice. **(A)** Forelimb (green) and hindlimb (purple) grip strength (male and female data combined) both decrease with age. Green and purple asterisks indicate significant differences among age groups for forelimb and hindlimb data, respectively. Bars represent mean ± SD, and numbers within bars indicate the number of animals measured. These data were obtained from a larger cohort of animals than those included in the ePhys and IHC experiments of this study. **(B)** Forelimb grip strength in male and female mice declines with increasing age (in months). **(C)** Hindlimb grip strength in male and female mice declines with increasing age (in months). **(D)** Correlation between forelimb and hindlimb grip strength across all age groups. Grip strength from fore and hind paws is strongly correlated, indicating a similar decline pattern. ***p < 0.001 and ****p < 0.0001 by two-way ANOVA; *p*-values in **(B,C)** represent Pearson’s correlation. Animal sample sizes for panels A–D are as follows: Forelimb: 85 young (42 females and 43 males), 81 middle aged (45 females and 36 males), and 168 old (75 females and 93 males); Hindlimb: 90 young (44 females and 46 males), 73 middle aged (40 females and 33 males), and 149 (66 females and 83 males). See Supplementary Table 1 for the correlation and regression information.

### 2.5 Confocal microscopy and analysis of channel clusters

#### 2.5.1 Confocal imaging and LCA determination

Images were obtained using an Olympus Fluoview 1000 confocal microscope with a 60x oil-immersion objective in 0.3 μm steps. *α*-MNs were differentiated in the medial and lateral grey matter of the ventral horn through visualization of the nucleolus, visible clusters expressing ChAT reactivity, and visible clusters expressing either SK2 or SK3 reactivity. Largest cross-sectional area (LCA) was determined using visualization of the z-stacks. Regions of interest (ROIs) were obtained using Fluoview software and measured. Analysis was blinded to both sex and age group throughout the study.

#### 2.5.2 3D cluster analysis

ROIs were generated for each z-stack using custom Python code developed and validated by members of the Neuro Engineering, Rehabilitation, & Degeneration (NERD) lab (Highlander et al., 2025). The algorithm uses edge detection to automatically outline cells by identifying areas with sharp changes in pixel intensity. Fluorescence values range from 0 (darkest) to 255 (brightest), and large differences between a pixel and its neighbors signal the presence of a positively stained edge. These edges are then traced to define the cell boundary. The process is semi-automated, requiring manual verification to confirm that the outlined structure is a motor neuron. On average, about 50 ROIs were created per cell, and each one was manually reviewed to ensure only the soma was included. The analyzer validating ROIs was blinded to both animal sex and motor neuron type, which was later identified using immunolabeling and code-based classification. ROIs were then run through a 3D MATLAB analysis script (Highlander et al., 2025). MNs were only included if they met established minimum size criteria (volume of 3,000 μm^3^ or LCA of 300 μm^2^) based on prior studies (Muennich and Fyffe, 2004; Dukkipati et al., 2018).

### 2.6 Physical strength analysis

#### 2.6.1 Mouse grip strength measurement

All animals included in this study underwent forelimb and hindlimb grip strength measurements before tissue collection. Forelimb grip strength was measured using the BIOSEB grip strength meter (BIOSEB *In Vivo* Research Instruments, BIO-GS3) with the mouse’s fore paws attached to the metal T-shaped bar while being pulled by the mouse’s tail (Figure 1B). Hindlimb grip strength was measured using the BIOSEB grip strength meter with the mouse’s hind paws attached to the metal T-shaped bar while the fore paws were holding a suspended bar (Figure 1B). The mouse was then pulled by the tail for the measurement of hindlimb grip strength. A total of eight forelimb and the hindlimb grip strength measurements were taken and the average of the top 3 values for each grip strength was used. Using the average of the top 3 limited variability due to uncooperative mice during grip strength testing (Derave et al., 2003). Forelimb grip strength on the day of ePhys collection was used to check for correlations between age-related weakness and MN excitability.

### 2.7 Computational modeling

To investigate the sensitivity of MN’s input conductance (Gin) and capacitance relative to MN anatomical size, we developed a computational model based on the reconstructed 3D morphology of a mouse MN (Ascoli et al., 2007) (See section 3.4). The model morphology included complete dendritic branching, soma, axon hillock, and initial segment. The electrical properties of the model, its parameters, and validation against experimental data are published in Mousa (2022). Specifically, membrane resistance (R_m_) for soma, AH, and IS were set to 636.4 $\Omega .cm^{2}$ , while the dendritic membrane resistance was set to 12,728 $\Omega .cm^{2}$ . The specific membrane capacitance ( $C_{m}$ ) was set to 1 μF/cm^2^, and the specific axial resistance ( $R_{a}$ ) was set to 70 Ω.cm .

**Figure 4:**
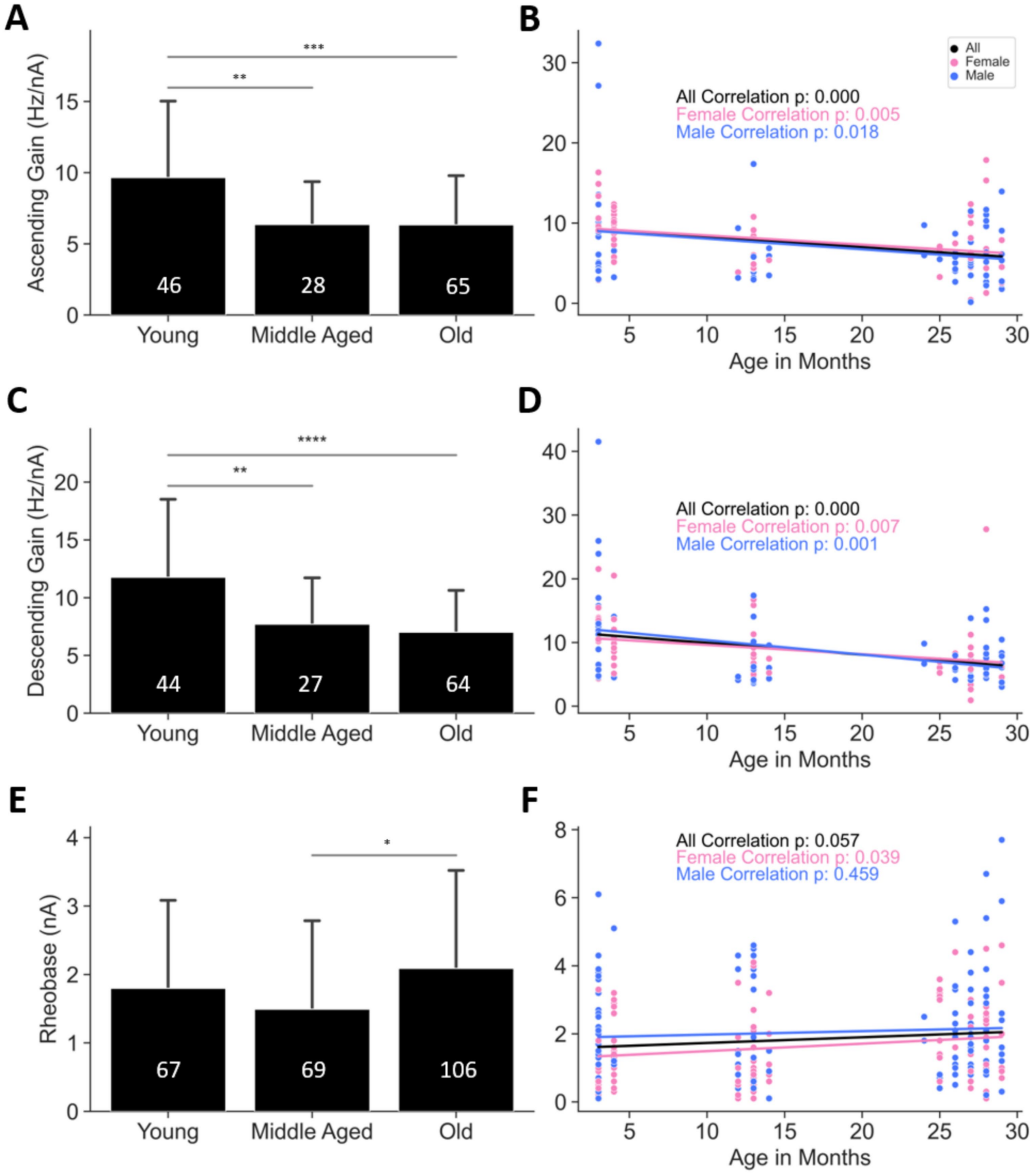
Motoneuron intrinsic excitability decreases with age. **(A,B)** Ascending gain and **(C,D)** descending gain both decrease in older mice. **(E,F)** Rheobase is elevated in old mice. *p < 0.05, **p < 0.01, ***p < 0.001, ****p < 0.0001 by one-way ANOVA; *p*-values in **(B,D,F)** are from Pearson correlations. Bars represent mean ± SD. Numbers within bars indicate the number of cells. The number of animals are as follows: **(A,B)** 30 young (16 females and 14 males), 22 middle aged (11 females and 11 males), and 47 old (21 females and 26 males); **(C,D)** 28 young (15 females and 13 males), 21 middle aged (10 females and 11 males), and 46 old (20 females and 26 males); **(E,F)** 43 young (22 females and 21 males), 42 middle aged (21 females and 21 males) and 59 old (28 females and 31 males). See Table 1 for the correlation and regression information.

In the simulations, input resistance (Rin) and time constant $\left(\tau_{0}\right)$ were measured using the same methods used in the experimental protocols. Specifically, Rin was measured by injecting long somatic hyperpolarizing current pulses of varying amplitudes while measuring the somatic voltage deflection from the resting membrane potential ( $V_{\text{rest}})$ . The time constant $\left(\tau_{0}\right)$ was measured using the graphical peeling method (Rall, 1969) applied on the somatic voltage traces returning to $V_{\text{rest}}$ after short hyperpolarizing currents pulses were injected somatically. The total cell capacitance was then calculated by dividing the time constant ( $\tau_{0})$ by the corresponding Rin.

To investigate the sensitivity of MN’s Gin and capacitance to the cell anatomical size changes, we measured these properties under the following conditions:

1. *Soma only*: The model cell had only a soma with no dendrites attached.
2. *Soma + dendrites*: The model cell with its original morphology was used, including the soma and dendrites.
3. *2x Soma + dendrites*: The somatic surface area of the original model was doubled. The dendritic surface area remained unchanged.
4. *3x Soma + dendrites*: The somatic surface area of the original model was tripled. The dendritic surface area remained unchanged.
5. *3x Soma + 2x dendrites*: The dendritic surface area of the original model was doubled, while its somatic surface area was tripled.
6. *2x Soma + 2x dendrites*: The dendritic and somatic surface areas of the original model were doubled.
7. *Soma + 2x dendrites*: The dendritic surface area of the original model was doubled without changing the somatic surface area.

Modifications to the surface area of any section were made by adjusting the respective diameter of that section.

### 2.8 Statistical analysis

Two types of analyses were employed in the present study: group analysis (represented by bar graphs) and temporal analysis (represented by scatter plots and regression lines).

#### 2.8.1 Group analysis

Young, middle aged, and old data were grouped and compared using one of the following methods: (1) One-way ANOVA with Tukey post-hoc test, used to assess the effect of aging on pooled (male and female combined) datasets, or (2) Two-way ANOVA with Tukey post-hoc test, used when examining the effects of aging and sex on separate male and female datasets. Grubb’s test was applied to detect outliers in each group. The number of motoneurons (MNs) included in each group is indicated inside the respective bar graphs unless otherwise stated. ANOVA analyses were conducted using IBM SPSS Statistics (version 29).

#### 2.8.2 Temporal analysis

Young, middle aged, and old data were plotted against age, and regression lines were computed alongside a Pearson correlation test. Unless otherwise noted, the Pearson correlation *p*-value is shown on each figure. Because these datasets were collected over multiple months, this analysis accounts for temporal effects.

All figures and Pearson correlations were generated in Python (packages used: pandas 1.5.3, pingouin 0.5.3, matplotlib 3.6.2, numpy 1.23.2, seaborn 0.12.2, statannotations 0.5.0, outliers 0.1, and scipy 1.10.0). For both analyses, statistical significance was defined as *p* ≤ 0.05. Asterisks indicate the level of significance as follows: *p ≤ 0.05, **p < 0.01, ***p < 0.001, and ****p < 0.0001.

## 3 Results

### 3.1 Grip strength progressively declines with age

**Figure 5:**
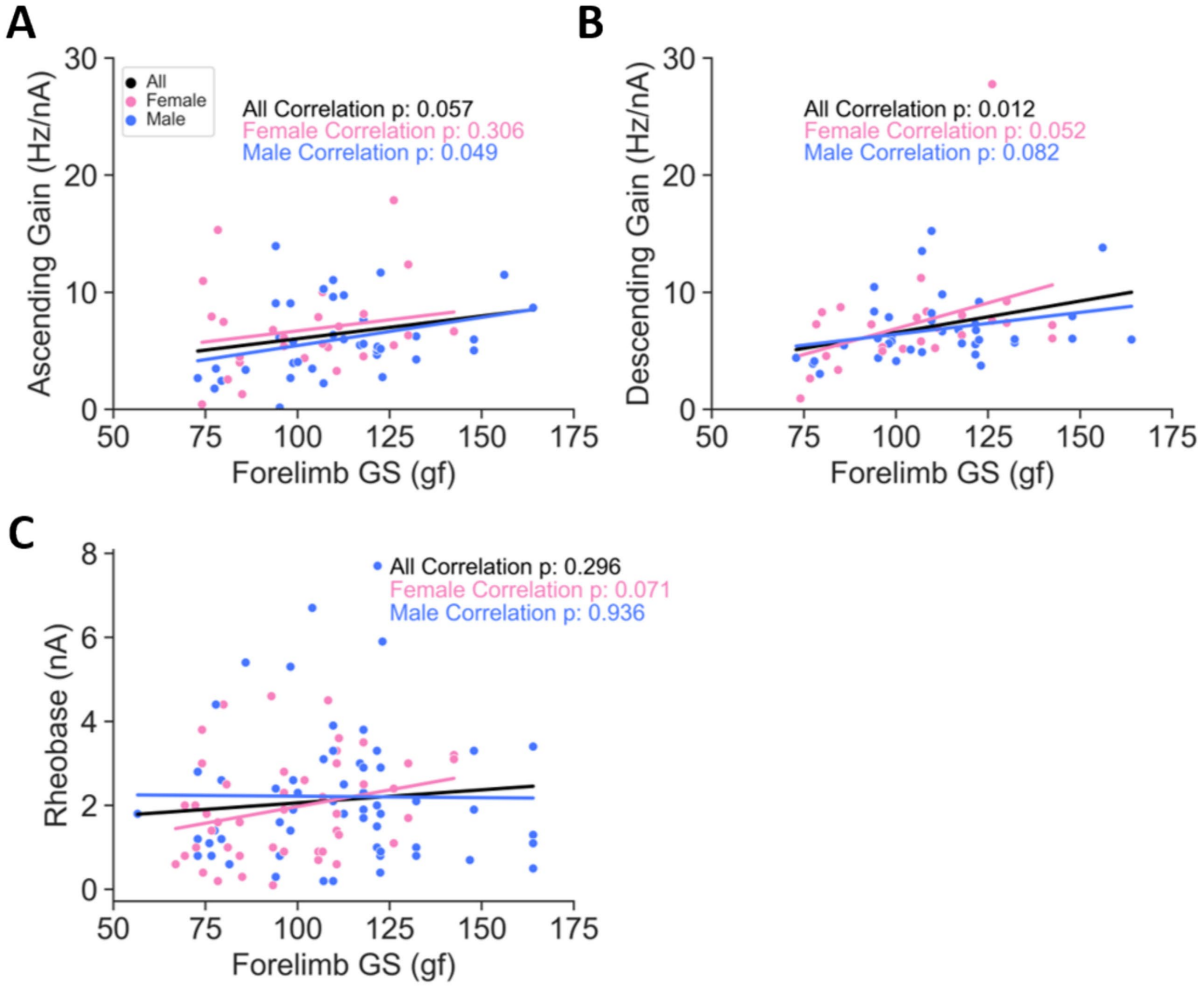
Motoneuron excitability is reduced in weaker old mice compared with stronger old mice. **(A)** Ascending gain is positively correlated with forelimb GS in males. **(B)** Descending gain is positively correlated with forelimb GS in females, with a trend observed in males. **(C)** Rheobase is not correlated with forelimb GS. All *p*-values in **(A–C)** are from Pearson correlation analyses. Data in this figure are from old mice only. The number of cells are as follows: **(A)** 65 old (27 females and 38 males); **(B)** 64 old (26 females and 38 males); **(C)** 106 old (47 females and 59 males). The number of animals are as follows: **(A)** 47 old (21 females and 26 males); **(B)** 46 old (20 females and 26 males); **(C)** 59 old (28 females and 31 males). See Supplementary Table 2 for the correlation and regression information.

To examine changes in force output from C57BL/6 mice during aging, we measured grip strength (in grams of force) in both hindlimbs and forelimbs in young, middle aged, and old mice. The group analysis results showed that mouse strength significantly decreases with age in both hindlimb and forelimb GS (*p* < 0.001 in all comparisons in Figure 3A), with hindlimb grip strength consistently lower than forelimb grip strength at both young and old ages (*p* < 0.001–0.0001, Figure 3A). Because the data from animals were collected over several months within each age group, a temporal analysis was performed to examine trends over time, with regression lines computed to determine the rate of progression of temporal changes. When separated by sex, both forelimb and hindlimb GS showed a statistically significant progressive reduction with age in both males and females (forelimb: *p* = 0.000, Figure 3B; hindlimb: *p* = 0.000, Figure 3C), and there was a strong correlation between the two measurements (*p* = 0.000, *r* = 0.606, power = 1.0, Table 2 and, Figure 3D), indicating that age-related weakness occurs across the entire motor system. However, because forelimb GS declines steadily throughout aging while hindlimb GS does not decline until old age (Figure 5A), forelimb GS appears more sensitive to age-related motor function decline in both sexes and was therefore used as the primary measure of motor strength in this study. Accordingly, our results align with previous work that, similar to humans, C57BL/6 mice experience an age-related decline in motor function, supporting their use as a model for studying age-related weakness (Arnold et al., 2017; Justice et al., 2014).

**Table 2:** The correlation between forelimb and hindlimb GS indicates that age-related declines in GS are highly correlated across limbs, underscoring the consistency of the aging effect on motor function.

Correlation test			
Distance		Pearson	
*r* = 0.633	*p* < 0.0001	*N* = 296	*r* = 0.606
		*p* < 0.0001	Power = 1.0

### 3.2 Age analysis—intrinsic MN excitability declines with advancing age

**Figure 6:**
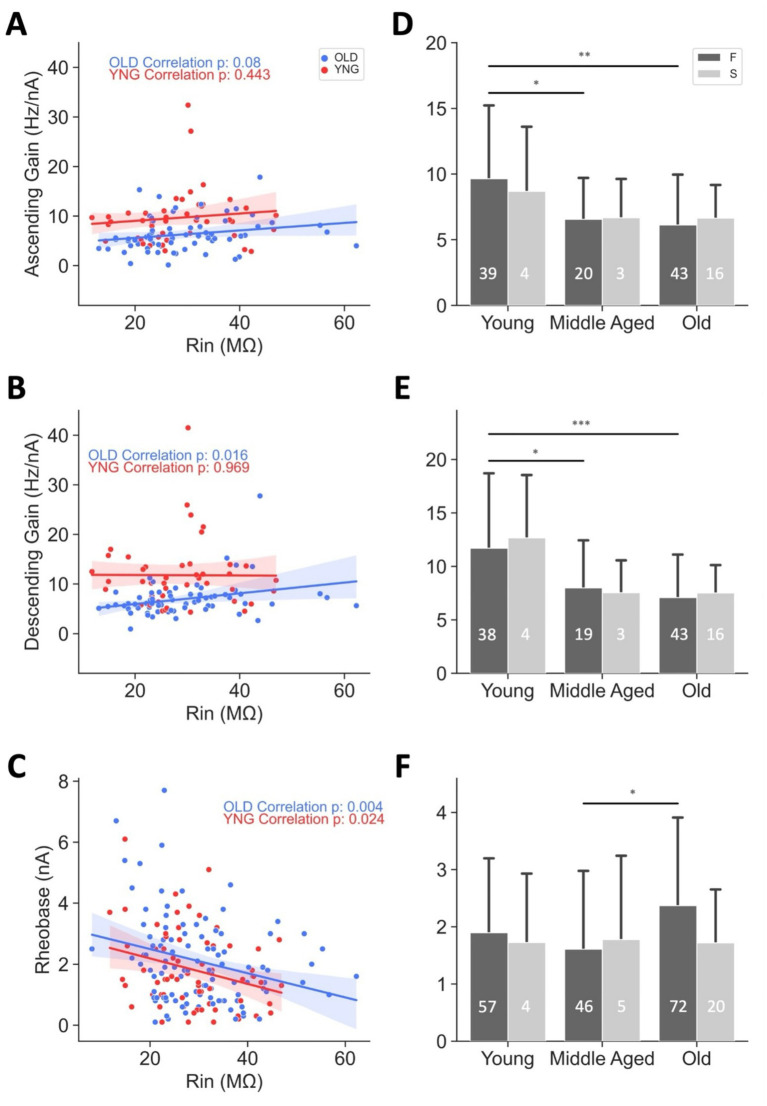
Large MNs are most affected by aging-related changes in excitability. Ascending gain versus *R*_in_ **(A)** and versus age (cells were split into slow and fast based on their AHP ½ decay, **D**) Descending gain versus *R*_in_ **(B)** and versus age by cell type **(E)**. Rheobase versus *R*_in_ **(C)** and versus age by cell type **(F)**. All *p*-values in **(A–C)** are from Pearson correlations, and shading indicates the 95% confidence interval. See Supplementary Tables 3–5 for the sample sizes, correlation, and regression information.

To assess MN intrinsic excitability with age, we measured the ascending and descending FI gains as well as rheobase of MNs across the three age groups. Both FI gain measures were significantly decreased in middle aged and old MNs compared to young MNs (Figures 6A,C; Table 1), whereas rheobase was increased in old compared to middle aged MNs (Figure 4E; Table 1). When sexes were studied separately, male and female MNs exhibited statistically significant progressive reduction in ascending and descending FI gains with age (*p* < 0.000–0.018, blue and pink traces in Figures 4B,D and Table 1), whereas rheobase progressively increased with age only in female MNs (*p* = 0.039, pink trace in Figure 4F and Table 1). Collectively, these results indicate that spinal MNs exhibit age-related excitability decline in both male and female mice, with female MNs exhibiting more pronounced changes, including increased rheobase and reduced FI gains, whereas male MNs demonstrate a reduction in FI gains only.

### 3.3 Strength analysis—MN reduced excitability underlies age-related weakness

**Figure 7:**
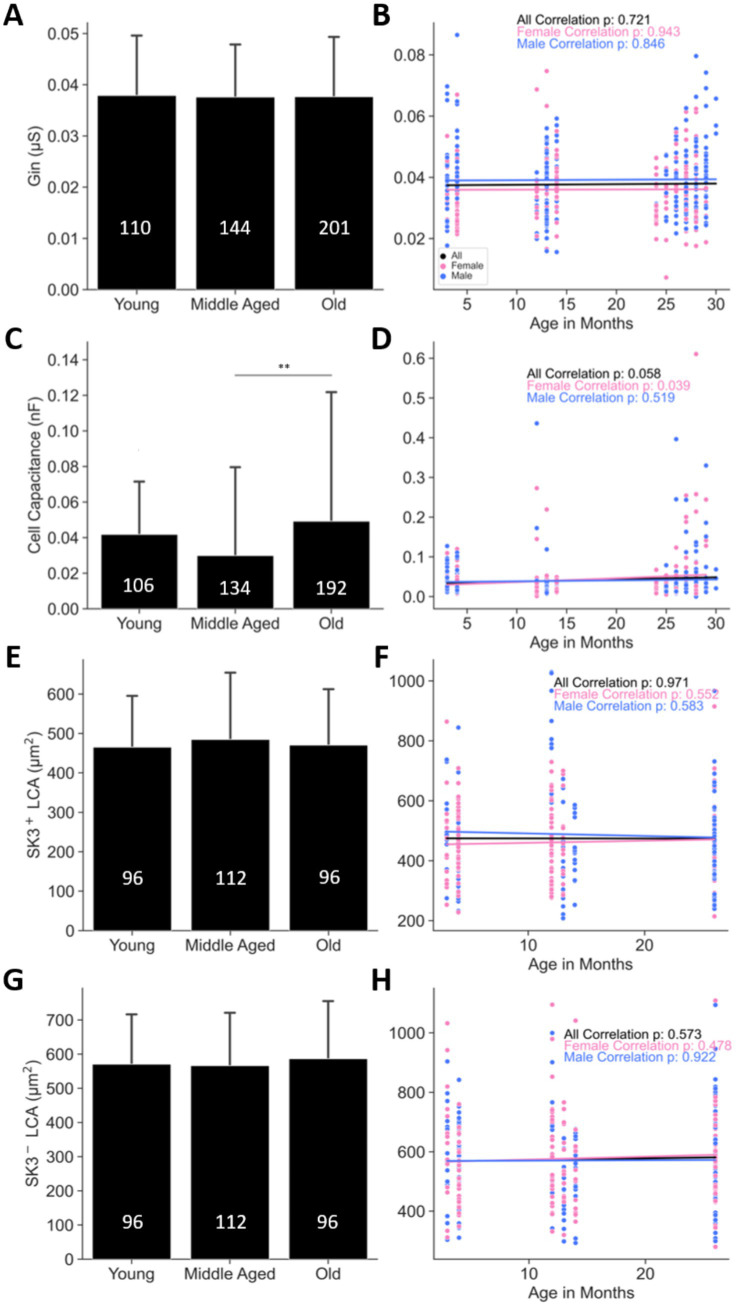
Motoneuron soma size remains unchanged with age, as demonstrated by electrophysiological and IHC measurements. **(A,B)** Input conductance (G_in_), and **(C,D)** cell capacitance measured in motoneurons from male and female mice across different ages. **(E,F)** Largest cross-sectional area of SK3^+^ labeled motoneurons, and **(G,H)** largest cross-sectional area of SK3^−^ labeled motoneurons from male and female mice across ages. Combined-sex data are shown on the left **(A,C,E,G)**, while data separated by sex are shown on the right **(B,D,F,H)**. *p* < 0.01 by one-way ANOVA; *p*-values in **(B,D,F,H)** are from Pearson correlations. Bars represent mean ± SD. The numbers within the bars indicate the number of cells. The number of animals are as follows: **(A,B)** 60 young (28 females and 32 males), 66 middle aged (34 females and 32 males), and 91 old (40 females and 51 males); **(C,D)** 59 young (27 females and 32 males), 66 middle aged (34 females and 32 males), and 91 old (40 females and 51 males); **(E–H)** 6 young (3 females and 3 males), 7 middle aged (4 females and 3 males), and 6 (3 females and 3 males). See Tables 1, 3 for the correlation and regression information.

To directly assess whether MN reduced excitability underlies age-related weakness, the ascending and descending FI gains and rheobase of MNs were analyzed in old mice alongside their forelimb GS measured on the day of electrophysiological experiments. For ascending FI gain, the overall correlation across all MNs was marginally significant (*p* = 0.057, black trace in Figure 5A and Supplementary Table 2); however, a significant positive correlation was observed specifically in male MNs (*p* = 0.049, blue trace in Figure 5A and Supplementary Table 2). These results suggest that in male mice, greater GS is associated with increased ascending gain, indicating that MN excitability is lower in weaker old male mice compared to their stronger counterparts. Similarly, descending FI gain demonstrated a significant positive correlation with forelimb GS across all MNs (*p* = 0.012, black trace in Figure 5B and Supplementary Table 2), further supporting a relationship between increased GS and enhanced descending gain. In contrast, rheobase did not exhibit a significant correlation with forelimb GS, as indicated by non-significant *p*-values for the entire group (*p* = 0.296, black trace in Figure 7C and Supplementary Table 2) and for both female (*p* = 0.071, pink trace in Figure 5C and Supplementary Table 2) and male (*p* = 0.936, blue trace in Figure 5C and Supplementary Table 2) MNs. These findings suggest that rheobase is relatively unaffected by variations in GS. In summary, these findings indicate that MN reduced excitability, as reflected in FI gain measures, directly correlates with age-related weakness in both sexes.

### 3.4 MN type analysis—fast MNs are the most affected by excitability reduction with age

Next, to identify the MN type most affected by age-related excitability changes, excitability metrics from young and old MNs were analyzed in relation to cell Rin and comparisons were made to determine whether distinct patterns emerged across different MN types (Figures 6A–C). To prevent visual overcrowding and extensive overlap among data points from the three age groups, only the young and old datasets (representing the two ends of the age spectrum, which demonstrated the most pronounced age-related differences in excitability, as illustrated in Figures 4A,B) were plotted in Figures 6A–C.

**Figure 8:**
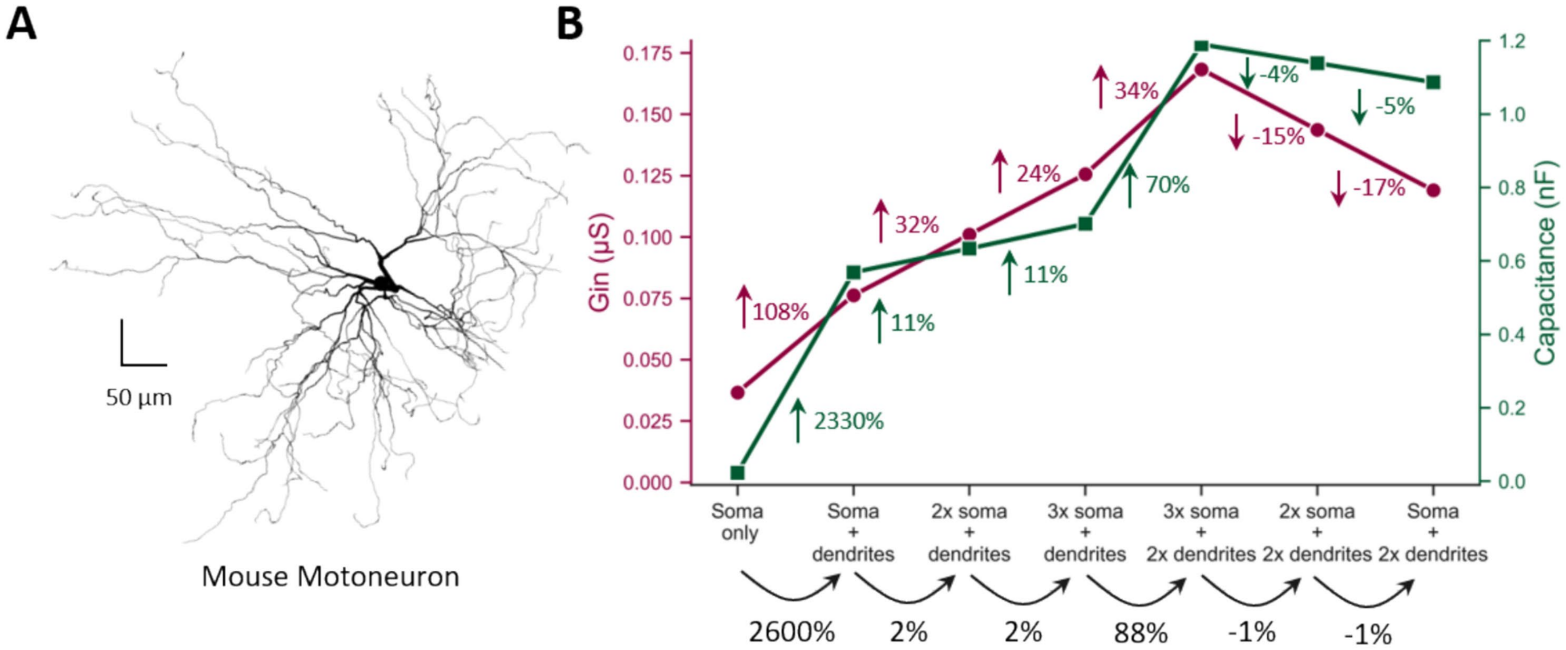
Sensitivity of MN G_in_ and cell capacitance to morphological changes. **(A)** The 3D reconstructed morphology of the mouse motoneuron model. **(B)** G_in_ (primary *y*-axis, maroon) and cell capacitance (secondary *y*-axis, green) plotted against various somatic and dendritic sizes (*x*-axis, black). Percent changes in G_in_, cell capacitance, and MN size at each step are indicated by maroon, green, and black arrows, respectively.

Rin based-comparisons revealed that ascending gain did not correlate with Rin in either young or old MNs; however, it was uniformly reduced with age across all MN types (see the lack of overlap in CI for most ranges of ascending gain between young and old MN data, Figure 6A and Supplementary Table 3). In contrast, descending gain showed no relationship with Rin in young MNs but demonstrated a significant positive correlation with Rin in old MNs (*p* = 0.016; blue trace in Figure 6B and Supplementary Table 3), suggesting that MNs with low Rin – typically large, fast MNs – experience a greater age-related reduction in descending gain than high-Rin (smaller, slow) MNs (the lack of overlap in CIs between large young and large old MNs in Figure 6B highlights this specific effect). As anticipated from the size principle, rheobase was negatively correlated with Rin in both young and old MNs; however, aging did not differentially affect rheobase across MN types, as evidenced by overlapping confidence intervals between young and old MNs (Figure 6C).

To further validate these findings, we classified MNs as fast or slow using AHP ½ decay duration (AHP ½ < 20 ms for fast MNs; >20 ms for slow MNs), a metric previously shown to accurately discriminate MN types (Gardiner, 1993). This analysis included all three age groups. However, because a larger percentage of MNs in the mouse spinal cord are fast, the sample size of slow MNs was smaller in this analysis. Consistent with the Rin-based results, fast MNs exhibited significant reductions in both ascending and descending gains between young and old ages (Figures 6D–E; Supplementary Table 4); however, a significant increase in rheobase between middle-aged and old groups was additionally observed in this analysis (Figure 6F; Supplementary Table 4). Although slow MNs displayed similar directional trends, these effects did not reach statistical significance, likely due to limited sample sizes (light bars in Figures 6D–F and Supplementary Table 5).

In summary, both Rin- and AHP-based analyses demonstrate that large, fast MNs are selectively vulnerable to age-related declines in excitability, whereas slow MNs show comparatively modest changes.

### 3.5 Female MN size increases with age

Here, we sought to examine the cellular changes underlying the reduced MN excitability with age. First, we assessed whether spinal MNs undergo structural changes with age by measuring input conductance (Gin) and cell capacitance—two properties that correlate with cell size—in young, middle aged, and old mice. Gin remained unchanged across age groups (Figure 7A; Table 1), with no correlation between Gin and age in either sex (blue and pink traces in Figure 7B and Table 1). In contrast, cell capacitance was significantly elevated in old mice, particularly when compared with middle aged animals (*p* < 0.01, Figure 7C and Table 1). When data were separated by sex, cell capacitance in female MNs showed a significant progressive increase (*p* = 0.039, pink trace in Figure 7D and Table 1), whereas male MNs did not exhibit any age-related changes (*p* = 0.519, blue trace in Figure 7D and Table 1). Given these conflicting Gin and cell capacitance results, we next investigated potential age-related changes in MN size using an independent technique: immunohistochemical (IHC) labeling. We identified and stained *α*-MNs with NeuN and Chat, to classify them into fast (F) and slow (S) types based on SK3 labeling (see Methods). Both SK3^−^ (F MNs) and SK3^+^ (S MNs) cells showed no change in somatic largest cross-sectional area (LCA) across ages (Figures 7E,G; Table 3), and neither sex showed correlation with age (blue and pink traces in Figures 7F,H and Table 3). These IHC findings support the Gin results, indicating that soma size remains unchanged.

**Table 3:** IHC SK2 and SK3 properties of α-MNs.

Parameter	Mean ± SD	One-way ANOVA	Pearson correlation		Regression slope	# of MNs	# of animals	Figures
SK3^+^ LCA (μm^2^)	YNG: 465.56 ± 130.55 MA: 485.08 ± 169.74 OLD: 470.76 ± 142.33	YNG-MA: *p* = 0.617 MA-OLD: *p* = 0.771 YNG-OLD: *p* = 0.969 *t*-statistic: 0.481	All: *p* = 0.971 ♀: *p* = 0.552 ♂: *p* = 0.583	All: *r* = 0.032 ♀: *r* = 0.032 ♂: *r* = 0.008	All: *m* = −0.034 ♀: *m* = 0.699 ♂: *m* = −0.842	YNG: 96 (♀: 48, ♂: 48) MA: 112 (♀: 56, ♂: 56) OLD: 96 (♀: 48, ♂: 48)	YNG: 6 (♀: 3, ♂: 3) MA: 7 (♀: 4, ♂: 3) OLD: 6 (♀: 3, ♂: 3)	Figures 7E,F
SK3^−^LCA (μm^2^)	YNG: 570.88 ± 146.53 MA: 566.56 ± 155.22 OLD: 586.86 ± 169.17	YNG-MA: *p* = 0.979 MA-OLD: *p* = 0.623 YNG-OLD: *p* = 0.761 *t*-statistic: 0.465	All: *p* = 0.573 ♀: *p* = 0.478 ♂: *p* = 0.922	All: *r* = −0.002 ♀: *r* = 0.047 ♂: *r* = −0.046	All: *m* = 0.568 ♀: *m* = 1.002 ♂: *m* = 0.142	YNG: 96 (♀: 48, ♂: 48) MA: 112 (♀: 56, ♂: 56) OLD: 96 (♀: 48, ♂: 48)	YNG: 6 (♀: 3, ♂: 3) MA: 7 (♀: 4, ♂: 3) OLD: 6 (♀: 3, ♂: 3)	Figures 7G,H
Total SK2 volume (μm^3^)	YNG: 39.89 ± 34.17 MA: 51.46 ± 26.22 OLD: 54.11 ± 39.13	YNG-MA: *p* = 0.0089 MA-OLD: *p* = 0.794 YNG-OLD: *p* = 0.001 *t*-statistic: 8.051	All: *p* = 0.003 ♀: *p* = 0.013 ♂: *p* = 0.004	All: *r* = 0.1715 ♀: *r* = 0.1619 ♂: *r* = 0.1880	All: *m* = 0.602 ♀: *m* = 0.531 ♂: *m* = 0.735	YNG: 176 (♀: 69, ♂: 107) MA: 131 (♀: 79, ♂: 63) OLD: 142 (♀: 63, ♂: 79)	YNG: 8 (♀: 4, ♂: 4) MA: 8 (♀: 4, ♂: 4) OLD: 8 (♀: 4, ♂: 4)	Figures 10A,B
# of SK2 clusters	YNG: 42.84 ± 25.19 MA: 58.01 ± 22.57 OLD: 55.61 ± 25.48	YNG-MA: *p* = 0.000 MA-OLD: *p* = 0.698 YNG-OLD: *p* = 0.000 *t*-statistic: 17.34	All: *p* = 0.000 ♀: *p* = 0.049 ♂: *p* = 0.001	All: *r* = 0.1976 ♀: *r* = 0.1340 ♂: *r* = 0.2873	All: *m* = 0.514 ♀: *m* = 0.287 ♂: *m* = 0.8636			Figures 10C,D
Total SK3 volume (μm^3^)	YNG: 38.00 ± 27.00 MA: 45.45 ± 31.92 OLD: 45.83 ± 26.82	YNG-MA: *p* = 0.045 MA-OLD: *p* = 0.993 YNG-OLD: *p* = 0.03 *t*-statistic: 4.339	All: *p* = 0.009 ♀: *p* = 0.227 ♂: *p* = 0.014	All: *r* = 0.1183 ♀: *r* = 0.0760 ♂: *r* = 0.1582	All: *m* = 0.348 ♀: *m* = 0.227 ♂: *m* = 0.451	YNG: 207 (♀: 103, ♂: 104) MA: 140 (♀: 70, ♂: 70) OLD: 146 (♀: 83, ♂: 65)		Figures 10E,F
# of SK3 clusters	YNG: 36.87 ± 17.71 MA: 43.06 ± 21.82 OLD: 41.45 ± 18.73	YNG-MA: *p* = 0.0097 MA-OLD: *p* = 0.757 YNG-OLD: *p* = 0.073 *t*-statistic: 4.910	All: *p* = 0.009 ♀: *p* = 0.217 ♂: *p* = 0.011	All: *r* = 0.1175 ♀: *r* = 0.0778 ♂: *r* = 0.1636	All: *m* = 0.234 ♀: *m* = 0.166 ♂: *m* = 0.295			Figures 10G,H

Because IHC labeling captures somatic but not dendritic size, we hypothesized that Gin primarily reflects soma size, whereas cell capacitance is more reflective of dendritic size. To test this, we developed a computational model of a mouse α-MN with a 3D-reconstructed morphology (Figure 8A) and systematically altered its somatic and dendritic dimensions independently to examine effects on Gin and cell capacitance. In this model, as in the literature (Dukkipati et al., 2018; Chalif and Mentis, 2022; Cullheim et al., 1987a; Cullheim et al., 1987b), the soma makes up ~4% of the total cell membrane surface area, while the dendrites account for ~96%. Accordingly, large increases in dendritic area (e.g., ~2,600 from step #1 to #2, and ~88% from step #4 to #5 in Figure 8B) produced only minimal increases in Gin (~108% and ~34%, respectively) but substantial increases in cell capacitance (~2,300% and ~70%, respectively). Conversely, small changes in soma size (+2% from steps #2 to #3 to #4, or −1% from step #5 to #6 to #7 in Figure 8B) led to more pronounced changes in Gin (2 × −3 × higher than the corresponding changes in cell capacitance). These findings indicate that cell capacitance is more sensitive to dendritic size, whereas Gin is more indicative of soma-specific changes. Consequently, the significant increase in cell capacitance in old female MNs (Figure 7D) strongly suggests dendritic enlargement—an effect that appears absent in old male MNs—thus reconciling the disparate Gin and cell capacitance findings in old mice. Collectively, these results indicate that while the soma size of both male and female MNs remains unchanged with age, female MNs exhibit an increase in dendritic size in old mice, which may contribute to their reduced excitability.

### 3.6 SK channel activation increases with age

Male MNs exhibit reduced excitability with age, yet they do not display the structural changes seen in females, suggesting that alternative ion channel dysfunctions may underlie this decline in excitability. To explore this possibility, single action potential (AP) properties were evaluated across three age groups. Our findings showed no significant alterations in AP properties (Table 1), indicating that fast sodium (Na^+^) and delayed rectifier potassium (K^+^) channels are unlikely to contribute to the reduced excitability of aged MNs. The lone exception was the resting membrane potential (RMP), which was hyperpolarized in aged MNs compared to middle-age (MA) MNs (Table 1). However, this anti-excitability change was driven by female MNs and was not observed in male MNs (RMP: −66.56 ± 7.17 mV in female MNs, −67.40 ± 8.04 mV in male MNs, Table 1). Since PICs have always been speculated to be affected by aging (Orssatto et al., 2021; Orssatto et al., 2023; Hassan et al., 2021; Guo et al., 2024), we examined ∆I recordings from triangle current ramps. However, ∆I measurements showed no significant changes with age (Table 1), suggesting that motoneuronal PICs remain unchanged.

**Figure 9:**
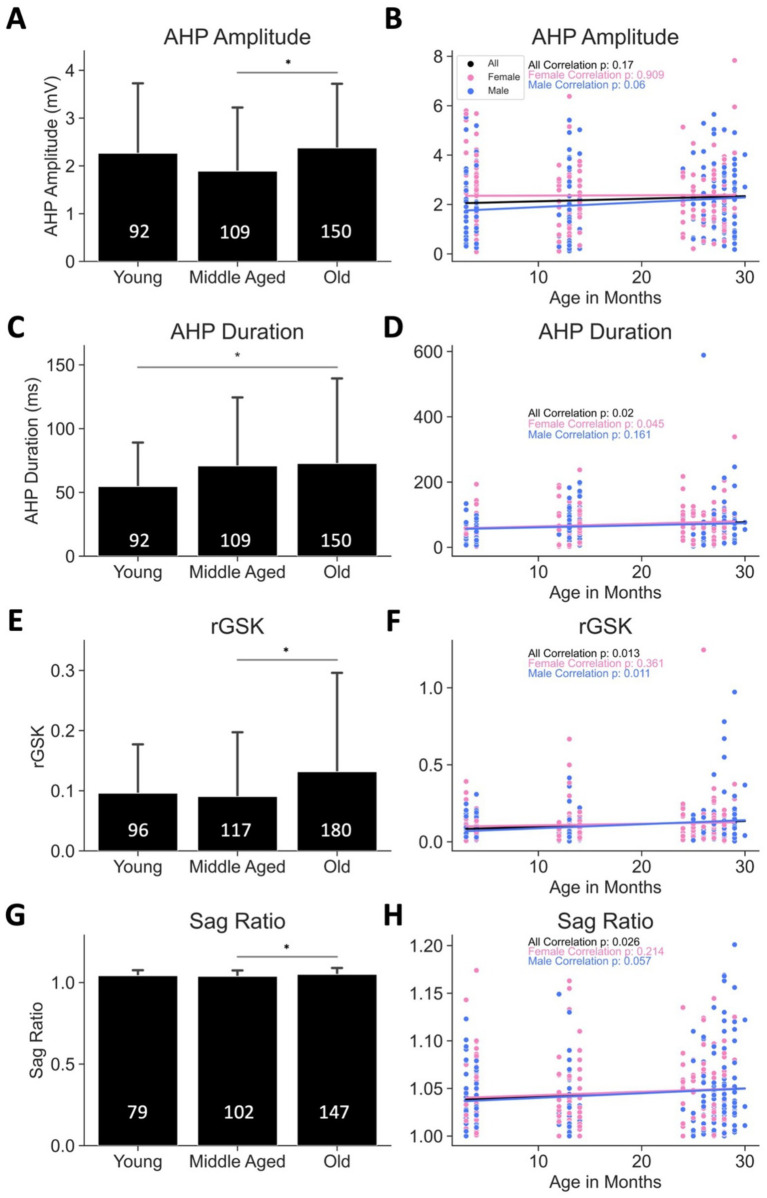
Increased SK channel activity with age. **(A,B)** AHP amplitude is higher in old mice. **(C,D)** AHP duration is prolonged in old mice. **(E,F)** Relative SK conductance is elevated in old mice. **(G,H)** Sag ratio is higher in old mice. *p < 0.05, **p < 0.01 by one-way ANOVA. *p*-values in **(D,F,H)** are from Pearson correlations. Bars represent mean ± standard deviation, and the numbers inside the bars indicate the number of cells. The number of animals are as follows: **(A,B)** 57 young (29 females and 28 males), 61 middle aged (33 females and 28 males) and 79 old (37 females and 42 males); **(C,D)** 57 young (29 females and 28 males), 61 middle aged (33 females and 28 males) and 79 old (37 females and 42 males); **(E,F)** 52 young (24 females and 28 males), 60 middle aged (33 females and 27 males) and 80 old (39 females and 41 males); **(G,H)** 56 young (26 females and 30 males), 63 middle aged (33 females and 30 males) and 90 old (39 females and 51 males). See Table 1 for the correlation and regression information.

Contrastingly, afterhyperpolarization (AHP) properties, mediated by small conductance calcium-activated potassium (SK) channels, exhibited heightened activity in aged MNs relative to both young and MA MNs. Specifically, AHP amplitude, AHP duration, and rGSK were all elevated in aged MNs (Figures 9A–F; Table 1; Figure 1D). However, aged MNs also demonstrated a significantly increased sag ratio (*p* < 0.05 in Figure 9G; *p* = 0.026, black trace in Figure 9H; Table 1 and Figure 1C), with a more prominent effect observed in male MNs. Given that an increased sag ratio is indicative of enhanced I_h_, which depolarizes the membrane potential during the AHP phase, it is likely that the observed AHP amplitude and rGSK values in aged MNs underestimate the true reduction in magnitude of SK channels activation.

**Figure 10:**
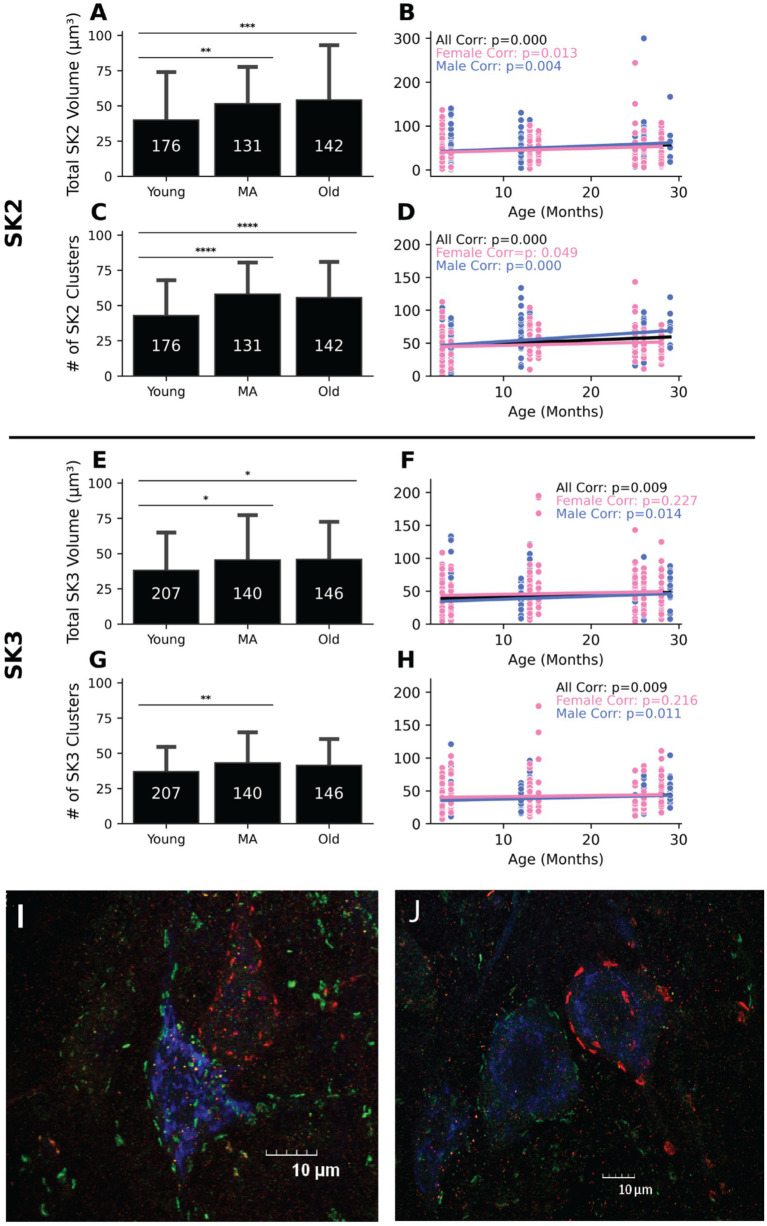
Soma SK2 and SK3 cluster volume and number increase with age. **(A,B)** Total SK2 volume increases with age. **(C,D)** SK2 cluster count increases with age. **(E,F)** Total SK3 volume increases with age. **(G,H)** SK3 cluster count increases in middle aged MNs. Combined-sex data are shown on the left **(A,C,E,G)**, while data separated by sex are shown on the right **(B,D,F,H)**. *p < 0.05, **p < 0.01, ****p < 0.0001 by one-way ANOVA. *p*-values in **(B,D,F,H)** are from Pearson correlations. Bars represent mean ± standard deviation, and the numbers within the bars indicate the number of cells. The number of animals for all figures are as follows: 8 young (4 female and 4 male), 8 middle aged (4 female and 4 male), and 8 old (4 female and 4 male). Representative images of SK2 (green) and SK3 (red) labeling in **(I)** young and **(J)** old mice. See Table 3 for the correlation and regression information.

To independently validate our electrophysiological findings, IHC staining of lumbosacral spinal cord sections from young, middle aged, and aged mice was performed, targeting SK2 and SK3 channel isoforms on the soma of the cells. Quantitative analyses were conducted to assess channel clustering properties, specifically total cluster volume and cluster count. The IHC results revealed an age-dependent increase in both the total cluster volume and the number of SK2 and SK3 clusters. In SK2-expressing MNs (fast-type, F MNs), both total cluster volume (Figure 10A, *p* < 0.01; Table 3) and cluster count (Figure 10C, *p* < 0.0001; Table 3) exhibited significant increases with age. These increases were driven by significant correlations in both male and female MNs (Figure 10B: *p* = 0.013 for males, *p* = 0.004 for females; Figure 10D: *p* = 0.000 for males, *p* = 0.049 for females, Table 3). Similarly, in SK3-expressing MNs (slow-type, S MNs), both total cluster volume (Figure 10E, *p* < 0.05; Table 3) and cluster count (Figure 10G, *p* < 0.05; Table 3) also increased with age. However, these changes were predominantly observed in male MNs (Figure 10F: *p* = 0.014 for males, *p* = 0.227 for females; Figure 10H: *p* = 0.011 for males, *p* = 0.216 for females, Table 3). Collectively, these findings support our ePhys data that age-related increases in SK channel expression occur in both slow (SK3^+^) and fast (SK2^+^) motoneurons across sexes, but are more pronounced in male MNs, which exhibit significant increases in both SK2 and SK3 clustering. In contrast, female MNs primarily show age-dependent increases in SK2 clustering.

### 3.7 SK shows type-specific changes with age

**Figure 11:**
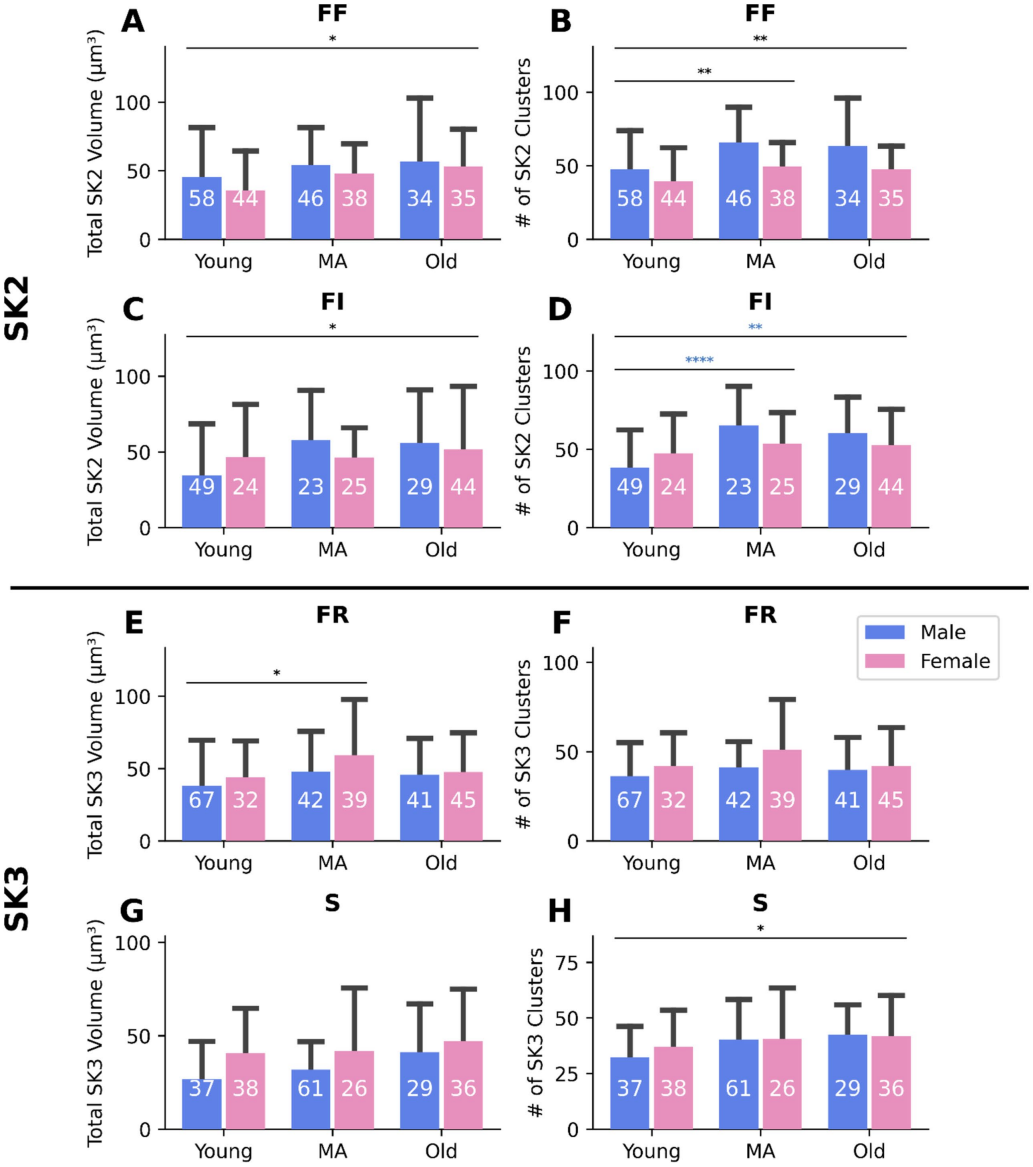
SK2 clusters show stronger age-related changes than SK3 clusters across MN types. **(A)** FF total SK2 volume is increased in old MNs. **(B)** FF SK2 number is increased in middle aged and old MNs. **(C)** FI total SK2 volume is increased in old MNs. **(D)** FI SK2 number is increased in male middle aged and old MNs. **(E)** FR total SK3 volume is increased in middle aged MNs. **(F)** FR SK3 number showed no change. **(G)** S total SK3 volume showed no change. **(H)** S SK3 number is increased in old MNs. *p < 0.05, **p < 0.01, ****p < 0.0001 by two-way ANOVA. Bars represent mean ± standard deviation, and the numbers within the bars indicate the number of cells. The number of animals for all figures are as follows: 8 young (4 female and 4 male), 8 middle aged (4 female and 4 male), and 8 old (4 female and 4 male). See Supplementary Table 6 for the correlation and regression information.

Our ePhys data indicate increased SK activity (via mAHP amplitude and duration, Figures 9A,C) in aged MNs, primarily in fast MNs (Figure 6). This was indirectly supported by a greater total cluster volume and number in SK2^+^ cells (F MNs) compared with SK3^+^ (S MNs) (Figure 10). To directly test this in type-identified cells, we classified MNs into S, FR, FI, and FF types by co-labeling for SK2, SK3, and OPN markers (See sections 2.4 and 2.5). Notably, FF and FI MNs showed a significant age-related increase in total SK2 cluster volume (Figures 11A,C; *p* < 0.05, Supplementary Table 6) and number (Figures 11B,D; *p* < 0.01, Supplementary Table 6). In contrast, FR and S MNs exhibited minimal age-related changes in total SK3 cluster volume (Figures 11E,G; Supplementary Table 6) and number (Figures 11F,H; Supplementary Table 6). These results directly confirm our ePhys findings that age-related SK upregulation occurs predominantly in fast MNs (FF and FI).

In summary, our electrophysiological and IHC data together suggest that SK channel activation is enhanced in aged MNs, primarily the fast types, contributing to their reduced intrinsic excitability.

## 4 Discussion

This study provides the first direct functional (Figures 9A–F) and structural (Figures 10, 11) evidence that upregulation of SK channels contributes to the age-related decline in MN intrinsic excitability in both male and female mice, further influenced by additional sex-specific cellular mechanisms. In terms of manifestations, both male and female MNs show reduced ascending and descending FI gains with age (Figures 4B,D); however, female MNs additionally exhibit an increased rheobase (Figure 4F). The cellular mechanisms underlying MN reduced excitability with age also differ between sexes. Both old male and female MNs experience increased expression of SK channels indicating an upregulation (Figures 10A–H). However, old female MNs further exhibit hyperpolarized RMP (Table 1) and cell capacitance (Figure 7D, indicative of dendritic membrane enlargement as suggested by our simulations in Figure 8), while old male MNs display a compensatory increase in I_h_ current (Figure 9H). Although ∆I measurements are an indirect assessment of PIC activation (Li and Bennett, 2003; Button et al., 2006), they do not indicate age-related changes in PICs (Table 1). When intrinsic excitability was correlated with functional force, a significant proportional relationship emerged: MN excitability was higher in old mice with higher grip strength (Figures 5A,B). Among all *α*-MN subtypes, fast MNs, FF and FI specifically, were the most affected by age-related excitability decline (Figures 6A,B, 11A–D). These findings emphasize the distinct aging pathways in male and female MNs and shed light on key motoneuronal mechanisms driving age-related weakness. This study offers valuable insights into the neuromuscular changes linked to functional decline in older adults, particularly in females, who have a higher likelihood of becoming frail with aging (Jenkins et al., 2022).

### 4.1 MN excitability decline: a common trait in males and females, more severe in females

Age-related weakness has traditionally been attributed to muscular atrophy, given its concurrent onset with aging (Clark and Manini, 2008). However, growing evidence indicates that muscle mass alone does not predict strength, prompting further investigations into the mechanisms underlying age-related weakness (Papadakis et al., 1996; Woodhouse et al., 2016; Becker et al., 2015; Delmonico et al., 2009). Recent studies have focused on the neuronal processes that mediate motor command transmission to muscles (Castro et al., 2023; Maxwell et al., 2018). The magnitude of motor command output is primarily determined by three factors: (1) the level of excitation, (2) the level of inhibition, and (3) the intrinsic excitability of MNs, all of which have been previously studied. For instance, Castro et al. (2023) and Maxwell et al. (2018) demonstrated that excitatory synaptic inputs are selectively and progressively lost from MN soma and dendrites with aging, thereby shifting the synaptic excitation-to-inhibition balance toward greater inhibition. In the present study, we examined the intrinsic excitability of MNs in the absence of synaptic inputs across aging and correlated these changes with functional forelimb and hindlimb grip strength in male and female mice. Although both male and female MNs exhibited an age-related decline in intrinsic excitability, the magnitude and underlying mechanisms differed by sex. Specifically, aged male and female MNs showed reduced FI gains with age, resulting in lower firing rates in response to a given input (Figures 4B,D). In females, however, this decline was further exacerbated by an age-related increase in rheobase, reducing MN recruitment (Figure 4F). Consequently, aged female MNs are recruited less frequently and fire at reduced rates compared with younger females, whereas aged male MNs maintain recruitment capacity but still exhibit lower firing rates when activated. These findings suggest that the decline in intrinsic MN excitability is more pronounced in females. These findings are also consistent with human studies demonstrating that older adults exhibit lower motor unit firing rates than younger adults (Wages et al., 2023; Orssatto et al., 2021; Orssatto et al., 2023; Hassan et al., 2021; Guo et al., 2023). Although reduced intrinsic excitability of MNs has previously been hypothesized in humans (Orssatto et al., 2021; Orssatto et al., 2023; Hassan et al., 2021), the present study provides direct experimental evidence supporting this idea and offers the first mechanistic insights into the motoneuronal ionic and membrane changes that underlie the sex-specific differences in age-related weakness.

Prior studies have investigated age-related changes in MN intrinsic excitability (Kalmar et al., 2009; Engelhardt et al., 1989; Morales et al., 1987); but their conclusions have varied, partly due to methodological variation. For instance, Kalmar et al. (2009) reported a reduction in FI gain (i.e., decreased MN excitability) with age; however, this study included only female rats, compared only young and old age groups (without a middle-aged cohort), and was based on a relatively small sample size (20 animals, 11 young and 9 old), limiting biological replication. In contrast, Morales et al. (1987) and Engelhardt et al. (1989) reported a reduction in rheobase (i.e., increased MN excitability) with age. These studies included both male and female cats, but data from both sexes were pooled without examining sex-specific differences, only adult and old groups were compared (no young group included), and sample sizes were again modest (22 animals, 12 young and 10 old). The present study addressed these limitations by including both male and female mice in larger numbers (264 animals for ePhys and IHC, 75 young, 81 middle-aged, and 108 old), enabling analysis of sex-specific effects Additionally, three age groups (young, middle-aged, and old) were incorporated to capture major staging of aging. The use of larger sample sizes and greater number of recorded cells enhanced statistical rigor and biological replication; however, this also contributed to the apparent variability in our datasets. To ensure that our conclusions are robust to this biological variability, we employed multiple complementary statistical approaches (bar graph–based group comparisons and scatter plot–based analyses), as well as independent experimental techniques (electrophysiology and IHC). The concordance of results across these analytical and experimental approaches provides strong validation of our findings and supports the robustness of our conclusions. Furthermore, earlier studies (Kalmar et al., 2009; Engelhardt et al., 1989; Morales et al., 1987) relied solely on electrophysiological measures of excitability, without assessing ion channel mechanisms or relating MN properties to functional muscle strength. These mechanistic and functional aspects were incorporated in the present study. Notably, we found that intrinsic MN excitability correlates with grip strength (measured from the same animals in which the MN recordings were obtained) in aged animals. Specifically, MNs from weak aged male and female mice exhibited lower excitability than those from stronger aged counterparts, a relationship observed in FI gains but not rheobase (Figures 5A,B). These findings parallel human studies indicating that older adults with weakness exhibit lower motor unit firing rates than their non-weak peers (Wages et al., 2023). Despite these methodological differences, our primary findings – namely the age-related reduction in intrinsic MN excitability and enhancement of the AHP amplitude and duration – are consistent with those reported by Kalmar et al. (2009). Additionally, we observed that old MNs exhibited an expanded Rin range extending toward higher values relative to young MNs (Figures 6A–C), consistent with the observations of Kalmar et al. (2009), indicating increased diversity within the MN population of aged animals. Importantly, our combined electrophysiological (Figures 9A–F) and IHC (Figures 10, 11) analyses implicate upregulation of SK channels as a contributing mechanism to this excitability reduction in both males and females and further reveal sex-specific changes – including changes in rheobase, RMP, and cell capacitance – predominantly in female MNs. Given the well-documented sex differences in human MN firing behaviors, muscle strength, and aging trajectories (Hwang and Park, 2022; de Jong et al., 2023; Lu et al., 2020; Jenz et al., 2023), elucidating the neuronal mechanisms underlying these physiological differences is essential for accurate and effective translation to human aging and disease. In this context, our male and female mouse data on MN properties and excitability across the lifespan provide valuable foundational insight for understanding MN-related neurodegenerative disorders, such as amyotrophic lateral sclerosis (ALS).

In the present study, we examined both adult lumbar MNs (IHC) and sacral MNs (electrophysiology) across the lifespan. In rodents, lumbar MNs innervate limb muscles, whereas sacral MNs innervate tail muscles; although these are distinct populations, both contribute to coordinated motor function. By combining electrophysiological analyses in sacral MNs with IHC assessments in lumbar MNs, our approach allowed us to determine whether the electrophysiological changes observed in sacral MNs extend to the functionally relevant lumbar MNs. Consistent with integrated lumbosacral motor control, we observed parallel, age-related declines in both forelimb and hindlimb grip strength (Figure 3; Table 2), suggesting that age-related weakness reflects a system-wide motor impairment. The age-related IHC changes identified in lumbar MNs correlate with the physiological alterations observed in sacral MNs, supporting a complementary pattern of adaptation. Likewise, the electrophysiological changes observed in sacral MNs correlate with the observed decline in forelimb and hindlimb grip strength. Taken together, these correlations support the concept of a broad and coordinated, age-associated decline in lumbosacral motor function (Lacava et al., 2024; Lacava et al., 2025; Etlin et al., 2010).

### 4.2 Fast MNs are more affected by excitability decline with age than slow MNs

*α*-MN subtypes differ in their force generation capacity (Burke et al., 1971). Our electrophysiology findings indicate that α-MNs’ intrinsic excitability declines unevenly with age. Specifically, fast MNs characterized by low Rin and short AHP ½ decay exhibit the greatest age-related decrease in excitability (Figures 6A,B), suggesting they are most affected by age-related decline. This was further supported by our IHC data on MN types, in which more pronounced SK changes were observed in fast, but not slow, MNs (Figure 11). In our MN electrophysiological classification, both Rin and AHP ½ decay were used to distinguish MN subtypes (Figure 6). However, because the majority of spinal MNs in mice are fast (Mathewson et al., 2012; Bloemberg and Quadrilatero, 2012), the AHP-based classification yielded in a relatively small sample of slow MNs. Given that Rin correlates with MN types and reliably differentiate MN subtypes in sharp electrode recordings (Highlander et al., 2020), Rin was additionally used as an indirect marker to validate the AHP-based analysis. Importantly, both approaches converged on the same result: aging preferentially impairs excitability in fast MNs. Consistently, both analyses showed that ascending FI gain was reduced in old versus young fast MNs (Figures 6B,D; Supplementary Table 4), and descending FI gain was selectively reduced in fast, but not slow, MNs with aging (Figures 6D,E; Supplementary Table 4). In addition, the AHP-based analysis revealed age-related increases in rheobase specifically in fast MNs (Figure 6F; Supplementary Table 4), further confirming the vulnerability of these cells in aging. Together, these findings confirm that fast α-MNs are selectively vulnerable to age-associated functional decline. Because FF MNs have a high force generation capacity, even a slight reduction in their firing rate may cause a substantial decrease in overall strength. This observation is consistent with human data simulations showing that a 3 Hz reduction in the motor unit firing rate of weak older adults corresponds to an 11–26% reduction in strength (Wages et al., 2023).

### 4.3 Gin and cell capacitance: distinct measures of MN size

Input conductance (Gin) and cell capacitance are commonly employed electrophysiological properties to estimate MN size (Bories et al., 2007; Quinlan et al., 2011). Although both measures are theoretically proportional to total membrane surface area, previous studies have reported paradoxical findings under identical experimental conditions in disease models characterized by MN hypertrophy. For instance, (Quinlan et al., 2011) observed an increase in Gin but no change in cell capacitance in G93A SOD MNs compared to wild-type (WT) MNs at postnatal day 10 (P10), a time point at which SOD MNs have an increase in soma size (Dukkipati et al., 2018). A similar discrepancy was observed in the present study, where Gin remained unchanged while cell capacitance increased with age (Figures 7A–D). This divergence suggests that Gin and cell capacitance do not equivalently reflect MN size. Our simulations resolved this contradiction by demonstrating that Gin is mainly influenced by somatic membrane surface area, whereas cell capacitance is more sensitive to dendritic membrane surface area (Figure 8B). Because Gin is derived from somatic current injection and voltage recording, it largely reflects somatic membrane properties. In contrast, cell capacitance is determined by the membrane time constant, which depends on charge equalization along dendritic pathways rather than on the site of current injection or recording (Holmes and Rall, 1992a; Holmes and Rall, 1992b; Holmes et al., 1992). Consequently, cell capacitance is a more reliable indicator of dendritic size changes rather than soma size alterations. Given the substantial variation in dendritic size among MNs (Cullheim et al., 1987a; Cullheim et al., 1987b), this may explain the notable inconsistencies in cell capacitance measurements across electrophysiological studies of MNs (Highlander et al., 2020). Accordingly, our findings suggest that the observed increase in cell capacitance in aged female MNs (Figure 7D) likely reflects dendritic hypertrophy, which is absent in aged male MNs. However, both Gin (Figures 7A,B) and LCA (Figures 7E–H) indicate that soma size remains unchanged across age groups, MN types, and sex, corroborating previous reports (Castro et al., 2023; Maxwell et al., 2018). Notably, prior studies have not systematically examined soma size in relation to sex or MN type (Castro et al., 2023; Maxwell et al., 2018), underscoring the novelty of the present analysis.

### 4.4 SK channel upregulation: a novel cellular mechanism contributing to age-related weakness

Our findings indicate that MNs undergo alterations in multiple ionic conductances in older MNs. Specifically, SK [which mediate outward I_SK_ current contributing to mAHP (Li and Bennett, 2007)] and HCN channels [which mediate I_h_ currents (Kiehn et al., 2000; Takahashi, 1990)] exhibit increased activation with age (Figure 9), while, surprisingly, ∆I [indicative of PICs, a composite current mediated by persistent Na, Ca, and SK channels (Li and Bennett, 2007)] appears unaffected (Table 1). However, it is important to note that this was based on ∆I measurements, which are an indirect assessment of PICs (see next section) (Kalmar et al., 2009). Because SK channels mediate the mAHP in MNs (Li and Bennett, 2007), their heightened activation with age amplifies the AHP’s amplitude and prolongs its duration. However, given the opposing effects of I_SK_ and I_h_ on the membrane potential during AHP (SK channels hyperpolarize while HCN channels depolarize), the increase in AHP amplitude was not as pronounced as the prolongation of its duration. This enhanced SK channel activation was independently confirmed by the increased clustering of SK2 and SK3 isoforms (Figure 10), whose cluster size and density directly reflect SK channel activity (Deardorff et al., 2013). Because SK channels are critical for the initiation and modulation of MN output (Mahrous and Elbasiouny, 2017; Mahrous and Elbasiouny, 2018), their increased activation with age likely reduces MN excitability and diminishes the motor command conveyed to muscles, thereby contributing to age-related weakness. In contrast, the rise in I_h_ current might serve as a compensatory mechanism to partially offset the effects of SK channel heightened activation in older MNs. As SK channels have not previously been implicated in aging, these data offer novel insights into the key motoneuronal mechanisms underlying functional decline in older adults.

### 4.5 PICs: an unresolved contributor

To our knowledge, motoneuronal persistent inward currents (PICs) have not been directly measured during aging or in the context of age-related weakness. Available data remain limited to indirect estimates based on ∆I measurements. Only two studies have assessed ∆I in relation to aging: the present study, which found no age-related changes in ∆I – suggesting that PIC amplitude may remain unchanged with age – and Kalmar et al. (2009), which reported an increased proportion of MNs exhibiting PIC-like behaviors in older animals, but did not compare ∆I values directly between young and old cells. Although ∆I has not been widely examined in this context, the related parameter ∆F has been extensively investigated in human studies. Several reports have demonstrated age-related declines in PIC activity (Orssatto et al., 2021; Orssatto et al., 2023; Hassan et al., 2021; Guo et al., 2024; Mohammadalinejad et al., 2024), as inferred from reduced ∆*F* values, across both upper (young: ~4.1–5.2 Hz; old: ~2.3–3.2 Hz) and lower (young: ~2.8–4.9 Hz; old: ~1.8–4.8 Hz) muscle groups (Orssatto et al., 2021; Orssatto et al., 2023; Hassan et al., 2021; Guo et al., 2024; Mohammadalinejad et al., 2024). Until motoneuronal PICs are directly measured (under voltage-clamp conditions) to determine whether their amplitudes change with age, the possibility of PIC changes contributing to aging-related excitability decline cannot be excluded. Nevertheless, our findings of SK channel upregulation and sex-specific mechanisms influencing MN excitability indicate that, even if PICs are involved, they are unlikely to represent the sole determinant of the age-associated reduction in intrinsic excitability.

## Data Availability

The raw data supporting the conclusions of this article will be made available by the authors, without undue reservation.
