# Supplementary material for: SK channel upregulation and sex-specific mechanisms drive spinal motoneuron reduced excitability with age
Source: Front Aging Neurosci. 2026 Feb 4;18:1687226. doi: 10.3389/fnagi.2026.1687226 (PMC12913407; doi:10.3389/fnagi.2026.1687226)
Supplement: Supplementary file 1 [file Supplementary_file_1.docx]

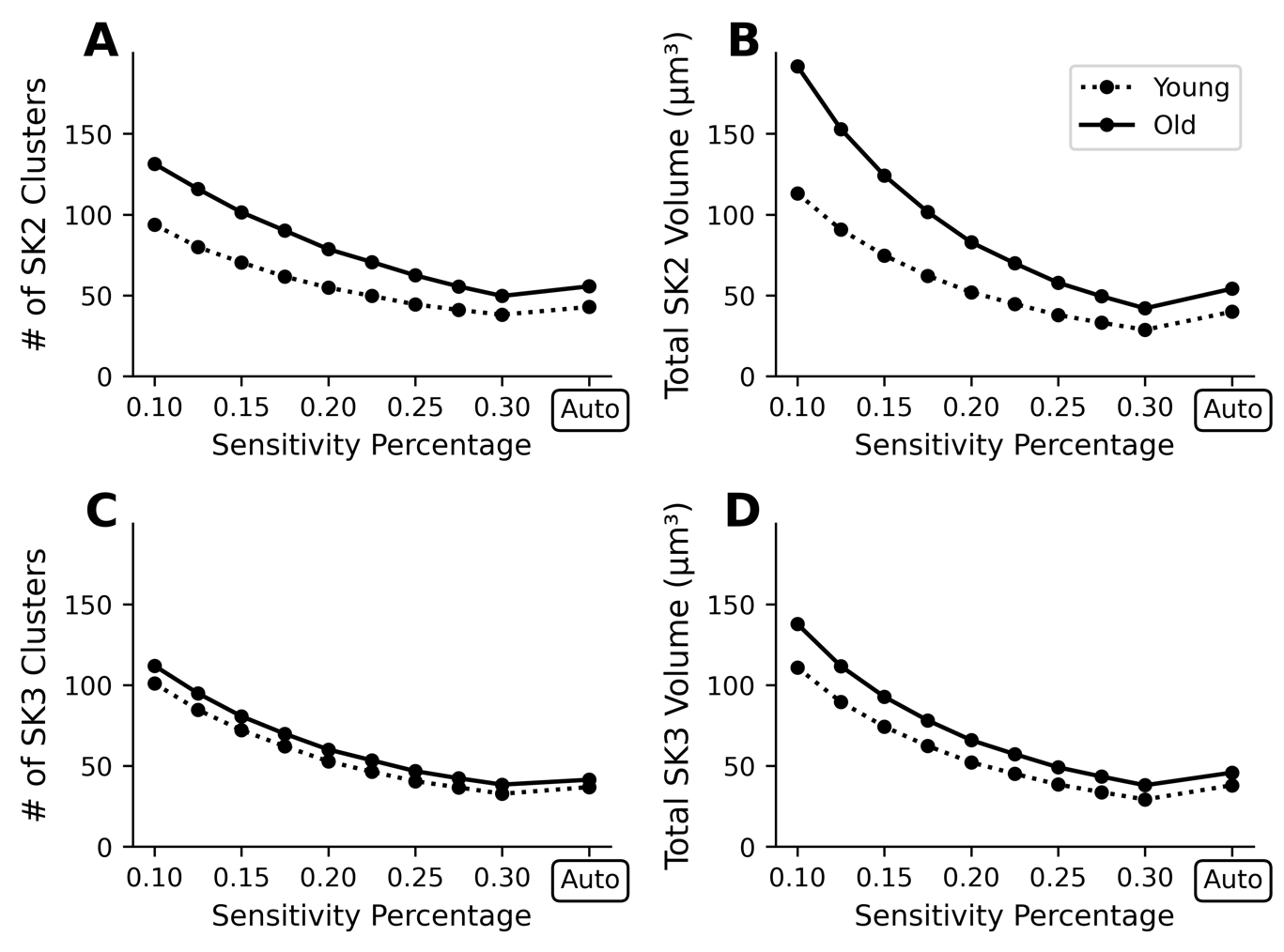


**Supplemental Figure 1: Age-related differences in SK cluster number and volume are preserved across a range of sensitivity thresholds.** **(A–B)** Number of SK2 clusters **(A)** and total SK2 cluster volume **(B)** are plotted across increasing sensitivity thresholds for cluster detection, comparing young (dotted line) and old (solid line) mice. **(C–D)** SK3 cluster number **(C)** and volume **(D)** show similar trends across thresholds. In all cases, old mice display consistently higher cluster counts and volumes than young mice, indicating that age-related increases in SK clustering are robust across detection parameters. The automatic threshold used in final analyses is shown at far right (“Auto”).

**Supplemental Table 1:** Age-related weakness - Correlation and regression information of the GS data relative to age.

| **Parameter** | **Mean ± SD** | **Pearson Correlation** | | **Regression**  **Slope** | **# of animals** | **Figure** |
| --- | --- | --- | --- | --- | --- | --- |
| **Forelimb GS (gf)** | YNG: 129.86 ± 23.62  MA: 104.66 ± 20.92  OLD: 94.46 ± 22.08 | ♀: p= 0.000  ♂: p= 0.000 | ♀: r= -0.530  ♂: r= -0.544 | ♀: m= -1.386  ♂: m= -1.511 | YNG: 85  (♀: 42, ♂:43)  MA: 81  (♀: 45, ♂: 36)  OLD: 168  (♀: 75, ♂: 93) | Fig 3B |
| **Hindlimb GS (gf)** | YNG: 102.33 ± 16.32  MA: 100.03 ± 20.27  OLD: 77.20 ± 19.59 | ♀: p= 0.000  ♂: p= 0.000 | ♀: r= -0.586  ♂: r= -0.523 | ♀: m= -1.343  ♂: m= -1.170 | YNG: 90  (♀: 44, ♂:46)  MA: 73  (♀: 40, ♂: 33)  OLD: 149  (♀: 66, ♂: 83) | Fig 3C |

**Supplemental Table 2:** Strength analysis - Correlation and regression information of the electrophysiology data relative to GS.

|  | **Pearson Correlation** | | **Regression**  **Slope** | **# of**  **MNs** | **# of**  **animals** | **Figure** |
| --- | --- | --- | --- | --- | --- | --- |
| Ascending Gain  (Hz/nA) | All: p= 0.057  ♀: p= 0.306  ♂: p= 0.049 | All: r= 0.238  ♀: r= 0.204  ♂: r= 0.322 | All: m= 0.039  ♀: m= 0.038  ♂: m= 0.048 | All: 65  ♀: 27  ♂: 38 | All: 47  ♀: 21  ♂: 26 | Fig 5A |
| Descending Gain  (Hz/nA) | All: p= 0.012  ♀: p= 0.052  ♂: p= 0.082 | All: r= 0.311  ♀: r= 0.385  ♂: r= 0.286 | All: m= 0.054  ♀: m= 0.088  ♂: m= 0.037 | All: 64  ♀: 26  ♂: 38 | All: 46  ♀: 20  ♂: 26 | Fig 5B |
| Rheobase  (nA) | All: p= 0.296  ♀: p= 0.071  ♂: p= 0.936 | All: r= 0.102  ♀: r= 0.266  ♂: r= -0.011 | All: m= 0.006  ♀: m= 0.016  ♂: m= -0.001 | All: 106  ♀: 47  ♂: 59 | All: 59  ♀: 28  ♂: 31 | Fig 5C |

**Supplementary Table 3:** MN type analysis - Correlation and regression information of the electrophysiology data relative to Rin.

|  | **Pearson Correlation** | | **Regression**  **Slope** | **# of**  **MNs** | **# of**  **animals** | **Figure** |
| --- | --- | --- | --- | --- | --- | --- |
| Ascending Gain  (Hz/nA) | YNG: p= 0.443  OLD: p= 0.080 | YNG: r= 0.116  OLD: r= 0.219 | YNG: m= 0.074  OLD: m= 0.075 | YNG: 46  OLD: 65 | YNG: 30  OLD: 47 | Fig 6A |
| Descending Gain  (Hz/nA) | YNG: p= 0.969  OLD: p= 0.016 | YNG: r= -0.006  OLD: r= 0.301 | YNG: m= -0.005  OLD: m= 0.108 | YNG: 44  OLD: 64 | YNG: 28  OLD: 46 | Fig 6B |
| Rheobase  (nA) | YNG: p= 0.024  OLD: p= 0.004 | YNG: r= -0.277  OLD: r= -0.275 | YNG: m= -0.042  OLD: m= -0.040 | YNG: 67  OLD: 106 | YNG: 43  OLD: 59 | Fig 6C |

**Supplementary Table 4:** MN type analysis – Descriptive and statistical information of the electrophysiology data relative to fast MNs.

|  | **Mean ± SD** | **One-Way ANOVA**  **(fast type)** | **# of**  **MNs** | **# of**  **animals** | **Figure** |
| --- | --- | --- | --- | --- | --- |
| Ascending Gain  (Hz/nA) | YNG: 9.66 ± 5.64  MA: 6.56 ± 3.23  OLD: 6.13 ± 3.88 | YNG-MA: p= 0.039  MA-OLD: p= 0.935  YNG-OLD: p= 0.002  t-statistic: 6.771 | YNG: 39  MA: 20  OLD: 43 | YNG: 28  MA: 17  OLD: 35 | Fig 6D |
| Descending Gain  (Hz/nA) | YNG: 11.71 ± 7.09  MA: 7.99 ± 4.56  OLD: 7.09 ± 4.06 | YNG-MA: p= 0.047  MA-OLD: p= 0.822  YNG-OLD: p= 0.0008  t-statistic: 7.531 | YNG: 38  MA: 19  OLD: 43 | YNG: 27  MA: 16  OLD: 35 | Fig 6E |
| Rheobase  (nA) | YNG: 1.90 ± 1.31  MA: 1.61 ± 1.38  OLD: 2.37 ± 1.55 | YNG-MA: p= 0.570  MA-OLD: p= 0.015  YNG-OLD: p= 0.153  t-statistic: 4.241 | YNG: 57  MA: 46  OLD: 72 | YNG: 40  MA: 33  OLD: 46 | Fig 6F |

**Supplementary Table 5:** MN type analysis – Descriptive and statistical information of the electrophysiology data relative to slow MNs.

|  | **Mean ± SD** | **One-Way ANOVA (slow type)** | **# of**  **MNs** | **# of**  **animals** | **Figure** |
| --- | --- | --- | --- | --- | --- |
| Ascending Gain  (Hz/nA) | YNG: 8.69 ± 5.68  MA: 6.67 ± 3.63  OLD: 6.65 ± 2.61 | YNG-MA: p= 0.039  MA-OLD: p= 0.935  YNG-OLD: p= 0.002  t-statistic: 0.607 | YNG: 4  MA: 3  OLD: 16 | YNG: 3  MA: 3  OLD: 14 | Fig 6D |
| Descending Gain  (Hz/nA) | YNG: 12.67 ± 6.78  MA: 7.54 ± 3.71  OLD: 7.52 ± 2.68 | YNG-MA: p= 0.047  MA-OLD: p= 0.822  YNG-OLD: p= 0.0008  t-statistic: 3.208 | YNG: 4  MA: 3  OLD: 16 | YNG: 3  MA: 3  OLD: 14 | Fig 6E |
| Rheobase  (nA) | YNG: 1.73 ± 1.39  MA: 1.78 ± 1.64  OLD: 1.72 ± 0.96 | YNG-MA: p= 0.570  MA-OLD: p= 0.015  YNG-OLD: p= 0.153  t-statistic: 0.006 | YNG: 4  MA: 5  OLD: 20 | YNG: 3  MA: 5  OLD: 16 | Fig 6F |

**Supplementary Table 6:** MN type analysis - Two-Way ANOVA analysis (with Tukey post hoc) of the immunohistochemistry data relative to cell type, age and sex

| **Cell type** | **Parameter** | **Age** | **Sex x Age** | **# of MNs** | **# of animals** |
| --- | --- | --- | --- | --- | --- |
| FF  cells | SK2  cluster volume  (Fig. 11A) | YNG-MA: p= 0.127  MA-OLD: p= 0.695  YNG-OLD: p= 0.016  t-statistic: 2.332 | No interaction | YNG: 102  (♀: 44, ♂: 58)  MA: 84  (♀: 38, ♂: 46)  OLD: 69  (♀: 35, ♂: 34) | YNG: 8  (♀: 4, ♂: 4)  MA: 8  (♀: 4, ♂: 4)  OLD: 8  (♀: 4, ♂: 4) |
|  | SK2  # of clusters  (Fig. 11B) | YNG-MA: p= 0.002  MA-OLD: p= 0.965  YNG-OLD: p= 0.007  t-statistic: 7.096 | No interaction |  |  |
| FI  cells | SK2  cluster volume  (Fig. 11C) | YNG-MA: p= 0.064  MA-OLD: p= 0.997  YNG-OLD: p= 0.034  t-statistic: 2.284 | No interaction | YNG: 73  (♀: 24, ♂: 49)  MA: 48  (♀: 25, ♂: 23)  OLD: 73  (♀: 44, ♂: 29) |  |
|  | SK2  # of clusters  (Fig. 11D) | YNG-MA: p= 0.0008  MA-OLD: p= 0.583  YNG-OLD: p= 0.001  t-statistic: 5.831 | ♂: YNG-MA: p= 0.000  ♂: YNG-OLD: p= 0.003 |  |  |
| FR  cells | SK3  cluster volume  (Fig. 11E) | YNG-MA: p= 0.018  MA-OLD: p= 0.412  YNG-OLD: p= 0.311  t-statistic: 2.291 | No interaction | YNG: 99  (♀: 32, ♂: 67)  MA: 74  (♀: 32, ♂: 42)  OLD: 86  (♀: 45, ♂: 41) |  |
|  | SK3  # of clusters  (Fig. 11F) | No age effect  t-statistic: 2.376 | No interaction |  |  |
| S  cells | SK3  cluster volume  (Fig. 11G) | No age effect  t-statistic: 3.030 | No interaction | YNG: 75  (♀: 38, ♂: 37)  MA: 81  (♀: 39, ♂: 42)  OLD: 86  (♀: 45, ♂: 41) |  |
|  | SK3  # of clusters  (Fig. 11H) | YNG-MA: p= 0.140  MA-OLD: p= 0.87  YNG-OLD: p= 0.045  t-statistic: 1.693 | No interaction |  |  |
